# An Event-Driven Self-Healing Routing and Topology Maintenance Mechanism for Surface-Deployed Wireless Sensor Networks in Ocean Environments

**DOI:** 10.3390/s26123915

**Published:** 2026-06-20

**Authors:** Lei Wang, Tzu-Ming Hsia, Chen-Wei Hsu, Pin-Yi Liu, Qian-Xun Hong

**Affiliations:** Department of Electrical Engineering, Feng Chia University, Taichung City 407102, Taiwan; leiwang@fcu.edu.tw (L.W.); wayne102493@gmail.com (C.-W.H.); pinyeee0601@gmail.com (P.-Y.L.); a0905889403@gmail.com (Q.-X.H.)

**Keywords:** surface-deployed wireless sensor networks, event-driven maintenance, self-healing routing, ocean monitoring, drift-aware networking, cluster-based routing, topology maintenance, conditional reclustering

## Abstract

Surface-deployed wireless sensor networks (WSNs) provide a flexible platform for ocean monitoring, but ocean-current-dominant marine forcing causes persistent topology evolution, backbone distortion, and route breakage. This paper proposes an event-driven self-healing routing and topology-maintenance mechanism for drift-prone surface WSNs. The design combines dual-threshold cluster-head handover, CH-HELP backbone repair, Node-HELP member reattachment, loop-free upstream reselection, and conditional global reclustering as a low-frequency corrective layer for long-term topology degradation. Unlike fixed-round reorganization, the proposed framework prioritizes local repair and triggers global refresh only when backbone quality persistently deteriorates. Simulations driven by Taiwan Strait current-dominant flow–wind data show that the full Proposed-Hybrid method reduces the CH-disconnection rate from 8.15% in DARCR to 5.15%, whereas the local-only configuration without conditional global reclustering yields 9.13%. Conditional global reclustering further suppresses late-stage topology degradation, reducing the final-third mean CH-disconnection rate from 16.32% to 8.51% and the late-stage 95th-percentile peak from 34.43% to 17.21%. DARCR remains competitive in some late-stage metrics because of its fixed-period global reorganization.

## 1. Introduction

Marine environmental monitoring is important for maritime safety, resource management, and disaster warning. Compared with conventional wide-area monitoring approaches, wireless sensor networks (WSNs) offer advantages in deployment flexibility and near-real-time data collection. However, marine environments are highly dynamic and difficult to maintain, posing significant challenges to sea-surface sensor networks in deployment, topology maintenance, and data transmission [1,2].

In terms of deployment, marine sensor networks can generally be classified into two types: anchored (or moored) and floating (or drifting) [3,4]. Anchored deployment constrains node displacement through mooring structures and therefore provides relatively high spatial stability. It is thus more suitable for long-term monitoring tasks at fixed locations. Under such an architecture, variations in node positions are limited, and the network topology remains relatively stable. Consequently, fixed neighbor relationships and transmission paths are easier to maintain, which is advantageous for applications requiring continuous long-term data collection from the same sea area while preserving stable reporting quality.

In practice, however, anchored systems still require additional consideration of water depth, anchoring equipment, deployment procedures, recovery operations, and long-term maintenance cost. In particular, in deeper waters, the length of mooring cables, structural loads, and deployment complexity all increase with depth, which further raises the costs of materials, vessel operations, and recovery [5]. Moreover, long-term exposure to the marine environment introduces problems such as biofouling, sensor contamination, and the need for periodic cleaning and maintenance, all of which further increase the long-term operational burden of the system [6].

By contrast, floating deployment avoids the need for extensive fixed infrastructure and offers benefits such as rapid deployment, high coverage flexibility, and easier expansion of the monitoring range. It is therefore better suited to scenarios requiring the rapid establishment of monitoring capability or adaptation to highly dynamic marine conditions. In particular, for large-area sea-surface monitoring, temporary mission deployment, or scenarios in which coverage must be adjusted according to changing sea conditions, floating architectures offer higher practical feasibility and application value [7].

However, the main challenge of floating deployment arises from its inherent mobility. Sensor nodes are continuously affected by ocean currents, tides, wind, and waves, causing dynamic changes in node positions, neighbor relationships, and routing paths. Over time, such drift may degrade network topology, disrupt backbone links, and reduce data transmission reliability.

If maintenance strategies designed for static environments are applied, the network may fail to respond promptly to disconnections caused by drifting. As a result, local failures may accumulate and lead to overall topology degradation. Therefore, maintaining network connectivity, backbone stability, and route recovery under continuous drifting remains a critical challenge in sea-surface WSNs [8].

In existing studies on cluster-based routing and topology maintenance, periodic updating based on fixed time or rounds is a common design paradigm. Protocols such as DEEC [9], EEUC [10], LEACH-MEEC [11], and DARCR [8] adopt scheduled maintenance to sustain network operation. However, such approaches rely on predefined intervals and may not respond effectively to dynamic topology changes. Periodic mechanisms rely on fixed schedules to perform maintenance, which easily creates identifiable temporal patterns and makes control-plane behavior highly regular. In scenarios where excessive regularity of control-plane timing is undesirable, such fixed maintenance schedules may impose additional limitations. However, this study does not directly quantify the effect of reduced control-timing regularity; rather, it treats this issue only as an architectural consideration related to maintenance scheduling [12].

Recovery actions are limited to scheduled updates, so disconnections occurring within a round may not be addressed immediately, increasing transmission delay and data loss risk.

Second, periodic mechanisms may cause excessive control overhead through global reorganization. To address local topology variations, periodic mechanisms often adopt network-wide reorganization. Even if most areas of the network are still functioning normally, all nodes may still be required to participate again in cluster election and reconfiguration. Such inflexible maintenance not only creates unnecessary control-packet overhead but also accelerates node-energy consumption.

Based on these observations, this study aims to reduce response delay and improve recovery efficiency by shifting from time-triggered global maintenance to event-driven local repair. Instead of performing network-wide reorganization at fixed intervals, the system initiates maintenance only when events such as link breakage, connection degradation, or topology changes occur. This approach reduces reliance on fixed schedules and shortens the impact duration of disconnections on data reporting.

From this perspective, the core question addressed in this study is as follows: without relying on fixed-time and network-wide synchronized reorganization, how can a sea-surface floating sensor network achieve self-maintenance, sub-step-level recovery, and long-term topology-quality preservation? To answer this question, this study focuses on three technical challenges.

First, determination of trigger timing. How can a trigger logic be designed based on node states and connection conditions, so that maintenance is initiated when necessary rather than merely at fixed times or round boundaries?

Second, feasibility of localized repair. When link breakage or local unreachability occurs, how can backbone recovery and member reattachment be completed through localized control signaling only, thereby avoiding unnecessary network-wide broadcasting and reorganization as in periodic schemes?

Third, topology adaptability under long-term drifting. Although localized repair can temporarily restore connectivity, cumulative drifting may still gradually elongate backbone routes, increase average hop count, and degrade overall topology quality. Therefore, how to balance sub-step-level localized repair with long-term backbone efficiency within a non-periodic architecture is also a central issue addressed in this work.

This study proposes an event-/condition-triggered routing-maintenance mechanism tailored for highly dynamic marine environments. The proposed approach is intended to overcome the limitations of conventional fixed-schedule reorganization strategies in terms of response timeliness, control cost, and long-term topology sustainability. The main contributions of this study are summarized as follows.

1. An event-/condition-triggered maintenance paradigm is proposed to replace fixed-round global refresh as the primary maintenance mechanism: The maintenance logic of sea-surface floating WSNs is transformed from fixed-schedule, network-wide synchronized reorganization into an abnormal-condition-driven, local-repair-first mode, enabling local recovery to be triggered within the same observation sub-step after abnormal conditions are detected, under the adopted discrete-time simulation framework. As a result, topology maintenance is performed only when necessary, thereby reducing maintenance gaps and reducing reliance on fixed-schedule network-wide reorganization.
2. A dual-threshold handover mechanism and a dual-layer self-healing repair framework are proposed to support event-driven maintenance: By combining energy thresholds, stability thresholds, CH-HELP, and Node-HELP, the proposed mechanism establishes a backbone-recovery and member-reattachment framework that can be triggered when local abnormalities are detected within the adopted sub-step-level framework, while also suppressing extra cost caused by frequent handovers, repeated HELP requests, and backbone oscillation. These components are designed as a coordinated maintenance framework rather than independent repair functions, so that CH role transfer, backbone recovery, and member reattachment can be handled under a unified event-driven logic.
3. A conditional global reclustering safety valve based on $\rho_{tail}(t)$ is introduced: Considering that long-term drifting may cause localized repair decisions to accumulate into backbone aging, route mismatch, and unreachable CH states, this study further introduces a topology-quality-driven conditional global reclustering strategy. The trigger monitors the long-hop ratio among reachable CHs and activates global reclustering only when sustained backbone elongation is observed. CHs that fail to report to the BS are reflected through CH-reachability and CH-disconnection statistics rather than used as an additional global-reclustering trigger. Unlike periodic global reorganization, this global operation is used only as a low-frequency safety valve when persistent backbone-quality degradation indicates that local repair is no longer sufficient.
4. The remainder of this paper is organized as follows. Section 2 reviews related work. Section 3 describes the network architecture, drift model, communication model, and residual-energy trigger definitions. Section 4 details the event-driven maintenance mechanism. Section 5 presents the simulation settings and performance evaluation. Section 6 concludes the paper and discusses limitations and future work.

## 2. Related Work

### 2.1. Cluster-Based Routing and Topology Control in Wireless Sensor Networks

Wireless sensor networks (WSNs) consist of a large number of low-power sensor nodes. Because node batteries are usually capacity-limited and difficult to replace, the design of energy-efficient routing protocols for prolonging network lifetime has long been one of the central research topics. In large-scale networks, flat routing architectures tend to incur broadcast storms and unbalanced energy consumption. By contrast, hierarchical and cluster-based routing can reduce control-plane overhead and improve energy-load distribution by allowing cluster heads (CHs) to aggregate and forward data [13]. Because clustering mechanisms typically involve multiple objectives and trade-offs, prior studies have also established classification frameworks to organize the characteristics and applicability of different clustering strategies systematically [14].

Cluster-based routing has been widely adopted in WSNs to improve energy efficiency and scalability. Classical protocols such as LEACH and HEED [15,16,17] focus on cluster-head selection and load balancing to prolong network lifetime. Recent surveys on LEACH-derived and energy-efficient clustering protocols further show that cluster-head selection, energy balancing, and inter-cluster routing optimization remain active research topics in WSN design [18,19]. Most of these works still focus primarily on energy efficiency, network lifetime, or general routing optimization, and they are mainly designed for static or low-mobility environments rather than event-driven topology maintenance under continuous sea-surface drifting.

In addition to routing, coverage and topology control have also been widely studied in energy-efficient WSN design. Existing work has explored coverage-preserving scheduling and topology-control mechanisms to improve network lifetime and energy efficiency [20,21,22,23].

When nodes are subject to continuous movement, the challenges extend beyond coverage and energy efficiency to include topology variation, connectivity maintenance, and timely recovery. The next subsection therefore reviews routing protocols designed for mobile and sea-surface drifting WSNs.

### 2.2. Protocols for Mobile and Sea-Surface Drifting WSNs

Compared with conventional static WSNs, mobile wireless sensor networks must additionally address changes in neighborhood relationships, cluster reorganization, and link stability caused by node mobility. In marine environments, factors such as ocean currents, tides, and wave action may continuously displace nodes or buoy platforms, thereby affecting backbone routes and reporting stability. Therefore, compared with ordinary static WSNs, sea-surface drifting sensor networks must simultaneously address node displacement, topology variation, and long-term connectivity maintenance.

To address node mobility, previous studies have proposed clustering-based routing protocols with mobility-aware mechanisms to improve cluster stability and reduce topology oscillation [24,25,26]. However, these approaches mainly target general mobile WSN scenarios and do not explicitly consider continuous drift in sea-surface environments.

To address the continuous-drift problem in sea-surface floating sensor networks, Wang and Hong proposed DARCR (Drift-Aware Routing and Clustering with Recovery) [8], which centers on drift-aware clustering and routing maintenance and aims to preserve low CH disconnection rates and low-delay transmission under continuously evolving topologies. DARCR consists of three main components: (1) an improved dynamic drift model to better capture node motion under realistic sea conditions; (2) a cluster-based route-construction mechanism intended to reduce disconnection and suppress delay; and (3) a self-recovery routing strategy that re-establishes communication links after disconnection and restores reporting capability. In addition, DARCR employs periodic network reorganization to refresh the topology and cope with backbone degradation induced by drift. Overall, DARCR provides a representative clustering and recovery baseline for sea-surface drifting scenarios and therefore serves as an appropriate benchmark for comparison in this work. However, its maintenance process still relies primarily on periodic reorganization, which contrasts clearly with the non-periodic, event-driven maintenance mechanism investigated in this study.

### 2.3. Self-Healing and Maintenance Mechanisms

During long-term operation, marine sensor networks are prone to disconnections caused by energy depletion, hardware failure, or link degradation. Therefore, self-healing and fault tolerance are essential for maintaining connectivity. Existing strategies generally involve a trade-off between global reorganization and local repair, where global refresh ensures topology consistency at high cost, while local repair reduces overhead cost but may lead to gradual topology degradation.

Event-driven mechanisms have been explored in WSNs, where threshold-based approaches enable adaptive responses to network conditions [27,28]. In addition, fault-tolerant clustering and topology-maintenance methods have proposed localized recovery strategies to preserve connectivity and reduce maintenance overhead [29,30,31]. However, balancing repair timeliness, control cost, and long-term topology quality remains a key challenge in dynamic environments.

### 2.4. Discussion and Research Positioning of This Work

This study therefore does not claim that a cluster-based architecture is universally optimal for all mobile-network scenarios; rather, it focuses on the maintenance trade-off of a reporting-oriented cluster/backbone structure under continuous sea-surface drift.

Existing studies have established foundations in cluster-based routing, mobility-aware design, and self-healing mechanisms for WSNs. In drifting marine environments, however, node movement is highly dynamic and uncertain, posing additional challenges to topology maintenance and connectivity. Drift prediction under ocean dynamics has also been studied [32,33,34], highlighting the uncertainty of node movement.

Most existing approaches rely on periodic maintenance as the primary strategy. Even DARCR [8], which targets sea-surface drifting scenarios, adopts periodic network reorganization to maintain connectivity. In contrast, this work proposes an event-driven, local-repair-first architecture, where global reclustering is used only as a supplementary mechanism when long-term topology degradation occurs.

Table 1 compares the proposed framework with DARCR in terms of maintenance strategy and recovery mechanisms. The results highlight that the proposed approach prioritizes event-driven local repair while limiting global reclustering to low-frequency, condition-triggered scenarios.

This study does not argue that cluster-based routing is universally preferable for all mobile-network scenarios. Rather, it focuses on a reporting-oriented sea-surface drifting network in which maintaining a sustainable cluster/backbone structure remains practically meaningful for multi-hop data delivery toward fixed base stations. Under this problem setting, the main issue is how to preserve reporting continuity, support localized recovery, and constrain long-term backbone degradation under continuous drift.

Reactive or opportunistic routing may be promising alternatives in other application settings and may also provide useful future extensions for the stability-related metrics considered in this work. However, the contribution of this study lies in the design of an event-/condition-triggered, local-repair-first maintenance architecture for the targeted sea-surface reporting scenario. The proposed framework integrates dual-threshold handover, CH-HELP, Node-HELP, route inheritance with upstream validation, loop avoidance, hop-growth control, and conditional global reclustering into a coordinated maintenance mechanism. Recent surveys on opportunistic routing and underwater IoT simulation environments also indicate that opportunistic forwarding and marine IoT routing remain important research directions under challenging communication conditions [35,36]. Nevertheless, their assumptions differ from the reporting-oriented surface WSN scenario considered in this study, where the main focus is maintaining a cluster/backbone structure under continuous drift.

## 3. Materials and Methods

### 3.1. Marine Environmental Sensing Deployment Model

Unlike static terrestrial wireless sensor networks, marine environments are highly dynamic and uncertain. To describe node behavior in the ocean more precisely, this study first establishes a standardized geometric and topological model for the monitoring network, node distribution, and base-station placement. The considered network architecture consists primarily of a large number of mobile floating sensor nodes and a small number of fixed-position base stations. The main notation and parameters used in this study are summarized in Table 2.

#### 3.1.1. Network Topology in the Taiwan Strait

This study considers the Taiwan Strait as the monitoring region. For mathematical derivation and simulation analysis, the target sea area is abstracted as a two-dimensional Cartesian plane. Let A denote the target monitoring area, whose length and width are L and W, respectively. In this study, the monitored region is set as a rectangular sea area of 100 km×100 km.

**Sensor-node set.** Let

$$N=\{n_{1},n_{2},\ldots ,n_{N}\}$$

denote the set of all sensor nodes, where N is the total number of nodes in the network. In the main simulation configuration, N = 1300 sensor nodes are randomly and uniformly distributed within the target area A. Additional node-number settings are evaluated later only for scalability analysis. The spatial position of node i at time t is represented by the two-dimensional coordinate vector

$$P_{i}\left(t\right)=\left(x_{i}\left(t\right),y_{i}\left(t\right)\right).$$

**Base-station set.** Let

$$B=\{BS_{1},BS_{2},\ldots ,BS_{M}\}$$

denote the set of data-collection centers. Considering that base stations in practical marine monitoring are typically installed along coastlines or on fixed offshore platforms, this study assumes that base stations are immobile. In the considered model, two fixed base stations are deployed at the trisection points along the long boundary of the monitoring region to improve initial data-reception coverage. Their coordinates remain unchanged throughout the simulation:

$$P_{BS}\left(t\right)=P_{BS}\left(0\right).$$

Under this topology model, all sensor nodes are required to report sensed data to any base station in the set B through either single-hop or multi-hop relaying. The node coordinates $P_{i}\left(t\right)$ vary continuously over time under the effect of the current-dominant flow–wind drift field, which also constitutes the key variable in the subsequent discussion of mobility modeling and routing disconnection.

#### 3.1.2. Mobility and Current–Wind Drift Model

In the marine drifting scenario considered in this study, sensor-node positions continuously change under a current-dominant flow–wind weighted drift field. The flow-field component is used as the primary drift component, while the wind-field component is incorporated as a secondary weighted term in the node-motion update. Let the position of node i at time t be $P_{i}(t)$. During each time step Δt, the node is subject to an equivalent horizontal drift velocity vector obtained from the weighted flow–wind field, whose components are denoted by $\left(u_{i}(t),v_{i}(t)\right)$. To quantify node drifting intensity, this study defines the instantaneous speed magnitude of node i as

(1) $$V_{i}\left(t\right)=\sqrt{u_{i}{\left(t\right)}^{2}+v_{i}{\left(t\right)}^{2}},$$

In this study, a discrete-time simulation framework is adopted, where one hour is defined as one round, and each round is further divided into six 10min sub-steps to characterize the responsiveness of event-driven maintenance. At the beginning of each sub-step, the system updates node positions and recomputes the pairwise distance matrix for subsequent connectivity checking and stability estimation. It should be noted that the event-driven maintenance discussed in this paper does not imply continuous-time, zero-latency real-time response; rather, it signifies that within this discrete-time framework, abnormalities can be detected and localized repairs can be triggered within the same observation sub-step in which they occur.

### 3.2. Communication Model and Energy-Aware Trigger Definitions

The two communication radii defined in this study, $R_{c1}$ and $R_{c2}$, are topology-level connectivity parameters used to distinguish different communication roles in the simulated cluster/backbone architecture. Specifically, $R_{c1}$ = 5 km represents short-range member-to-CH and intra-cluster control communication, whereas $R_{c2}$ = 20 km represents long-range backbone communication among CHs and between CHs and base stations. These values follow the prior DARCR simulation configuration and are used as binary connectivity radii for topology-maintenance evaluation.

The adopted communication model should therefore be interpreted as a controlled topology-level abstraction rather than a hardware-validated physical VHF propagation model for all sea-surface deployment conditions. A link is considered available when the corresponding Euclidean distance is within the assigned communication radius. This abstraction allows the proposed routing-maintenance mechanism to be evaluated under consistent connectivity assumptions. The influence of the backbone communication radius is further examined through the $R_{c2}$-sensitivity analysis in Section 5.7.2.

This study uses hop count to describe the routing level from a cluster head to a base station. Let $H\left(X\right)$ denote the hop count from node X to a base station, and let the base station itself be defined as $H\left(BS\right)=0$. If a node currently has no available upstream, its hop status is regarded as undefined or, equivalently, +∞. If cluster head C selects U as its upstream, then its hop count is

$$H\left(C\right)=H\left(U\right)+1.$$

In this study, the residual-energy state used for CH-handover triggering is modeled as a normalized packet-operation energy state rather than an absolute physical Joule-based radio-energy model. Each node is initialized with $E_{0}=5$ normalized energy units. A receive operation consumes $E_{RX}=0.0005$ units, an intra-cluster short-range transmission consumes $E_{TX-low}=0.0010$ units, and a longer-range transmission consumes $E_{TX-high}=0.0030$ units. These values are selected to preserve the relative operation-cost ordering $E_{RX}<E_{TX-low}<E_{TX-high}$. In this setting, receiving is assigned the lowest cost, intra-cluster transmission is used as the baseline transmission cost, and longer-range backbone or inter-cluster transmission is assigned a higher cost.

The residual-energy update is event-based: each receive or transmit operation deducts the corresponding normalized cost once. Packet-size-dependent energy, continuous distance-dependent path loss, idle energy consumption, retransmission cost, and MAC-layer contention are not explicitly modeled in this residual-energy update. Therefore, $E_{TRIGGER}=2.5$ and ΔE=1.5 are interpreted as normalized residual-energy thresholds for CH-handover control rather than absolute physical Joule values.

For the residual-energy trigger design, let $E\left(i\right)$ denote the residual energy of node i. For cluster-head handover, this study employs a normalized residual-energy threshold $E_{TRIGGER}$ as Gate A and a normalized residual-energy difference threshold ΔE as Gate B. When a cluster head C satisfies

$$E\left(C\right)<E_{TRIGGER},$$

it is considered to have entered a low-energy risk state. If there exists a candidate node n within the cluster such that

(2) $$E\left(n\right)\geq E\left(C\right)+\Delta E,$$

then a successor with a significant energy advantage exists, and the handover evaluation may be initiated to reduce the risk of abrupt cluster failure and to redistribute the cluster-head role before the current CH becomes critically depleted. Here, C denotes the current CH, n denotes a candidate successor, and ΔE is the minimum residual-energy advantage required for energy-triggered handover evaluation.

### 3.3. Stability and Disconnection Metrics

#### 3.3.1. Self-Stability

In a marine drifting environment, relative node motion may destabilize both cluster structure and backbone links. To quantify the drift stability of an individual node, this study defines self-stability $S_{self}$ as a monotonically decreasing function of speed magnitude. To prevent the interpretation of the stability threshold from being directly affected by the velocity unit, a reference speed scale $V_{ref}$ is introduced, and self-stability is defined as

(3) $$S_{self}\left(i,t\right)=\frac{1}{1+V_{i}\left(t\right)/V_{ref}},$$

When $S_{self}<S_{THRESHOLD}$, the node is regarded as being in an unstable state and should be preferentially considered for handover or backbone-route adjustment so as to reduce the risk of imminent disconnection.

The reference speed $V_{ref}$ is a fixed normalization constant rather than a time-varying node velocity. In this study, $V_{ref}$ is set to 1.0 m/s. The value is used as a fixed reference scale to keep the self-stability score dimensionless and comparable across simulation runs, rather than as a fitted or time-varying physical threshold. The instantaneous node speed *V_i_*(*t*) varies over time according to the current-dominant flow–wind drift field, whereas $V_{ref}$ only determines the scale of the self-stability score. Therefore, $S_{self}$ is used as a CH-level drift-intensity indicator for identifying whether the current CH is becoming unsuitable for the CH role.

#### 3.3.2. Upstream Validity and Disconnection State

For a cluster head C, let $U_{C}$ denote the currently selected immediate upstream of C. The upstream may be either a base station or another cluster head. In this study, $S_{up}(C)$ is defined as a local upstream-validity state that indicates whether the immediate upstream of C is currently reachable and available under the backbone communication-radius constraint. It does not recursively encode the validity of the entire upstream chain.

(4) $$S_{up}\left(C\right)=\left\{\begin{array}{ll} 1, & \;U_{c}\;\in B\;and\;d\left(C,U_{c}\right)\leq R_{c2},\\ 1, & U_{c}\in CH,\;U_{c}\;is\;alive,d\left(C,U_{c}\right)\leq R_{c2},\\ 0, & \mathrm{otherwise}. \end{array}\right.$$

Here, B denotes the set of base stations, CH denotes the set of cluster heads, $U_{C}$ is the immediate upstream selected by C, and $d(C,U_{C})$ is the Euclidean distance between C and its upstream. The first case states that a direct CH-to-BS upstream is locally valid only when the selected base station is within the backbone communication radius $R_{c2}$. Therefore, a base station is not treated as a valid upstream merely because the upstream identifier refers to a BS. The second case states that a CH-to-CH upstream is locally valid only when the upstream CH is alive and locally reachable within $R_{c2}$.

Although $S_{up}(C)$ is a local immediate-upstream state, it is evaluated and updated under a BS-outward validation order. CHs closer to a base station, or equivalently CHs with smaller hop levels, confirm their upstream validity before downstream CHs. This processing sequence is referred to as the BS-outward validation order in this study. Therefore, when a downstream CH selects an upstream candidate during CH-HELP, the candidate CH has already passed upstream-validity confirmation from the upper layer in the current validation pass.

Complete upstream-chain checking and routing-loop prevention are not embedded recursively in Equation (4). Instead, they are handled by the bounded upstream-chain validation described in Section 4.3. This validation is activated on demand during route inheritance, CH-HELP, and upstream reselection when path-level checking is required. It verifies hop-by-hop $R_{c2}$ reachability, upstream-CH survival, undefined upstreams, maximum validation depth, and possible routing loops.

At the beginning of each sub-step, if $S_{up}(C)=0$, the CH is regarded as having an invalid immediate upstream and enters the corresponding repair procedure. If $S_{up}(C)=1$, the CH has a locally reachable upstream next hop under the current BS-outward validation order. When route inheritance or repair decisions are involved, the upstream chain is further checked by the bounded upstream-chain validation mechanism.

#### 3.3.3. Event-Level Disconnection Definition

To distinguish newly occurred disconnections from persistent ones, this study records two disconnection states for each node or CH (collectively denoted as X): the previous-sub-step disconnection state $D_{prev}\left(X\right)\in \{0,1\}$ and the pre-repair disconnection state in the current sub-step $D_{now}\left(X\right)\in \{0,1\}$. Events are defined as follows:

1. new disconnection event (connect to disconnect): $D_{prev}\left(X\right)=0$ and $D_{now}\left(X\right)=1$;
2. persistent disconnection (disconnect to disconnect): $D_{prev}\left(X\right)=1$ and $D_{now}\left(X\right)=1$ (not counted repeatedly as a new event);
3. recovered event (disconnect to connect): $D_{prev}\left(X\right)=1$ and $D_{now}\left(X\right)=0$.

This design allows disconnection and recovery counts to be clearly recorded on an event basis, avoiding repeated counting of a long-lasting disconnection at every sub-step.

#### 3.3.4. Link Stability

In upstream selection and HELP-based recovery, this study uses link stability to quantify the likelihood that the connection between two nodes will remain reachable over the short term. For any two nodes A and B, the relative speed magnitude is first defined as

(5) $$V_{rel}\left(A,B\right)=\sqrt{{\left(u_{A}-u_{B}\right)}^{2}+{\left(v_{A}-v_{B}\right)}^{2}},$$

Given a speed-scale constant $V_{0}$, the velocity-based stability term is defined as

(6) $$S_{link}\left(A,B\right)=\frac{1}{1+V_{rel}\left(A,B\right)/V_{0}},$$

The speed-scale constant $V_{0}$ is fixed at 0.5 m/s in the simulation. It normalizes the relative-speed term in the link-stability calculation and is not a time-varying velocity value. The value is selected as a fixed relative-speed scale so that pairwise velocity differences are mapped to a bounded stability score used consistently in upstream selection and CH-HELP repair.

#### 3.3.5. Upstream-Selection Score and Hop-Increase Constraint

For a cluster head C, a candidate upstream U must have a valid path to a base station, that is, $H\left(U\right)$ must be known and finite. This study defines an upstream-selection score function that combines link stability and hop penalty:

(7) $$Score\left(C\to U\right)=w_{1}\;S_{link}\left(C,U\right)-w_{2}\;H\left(U\right),$$

where $w_{1},w_{2}>0$ are weighting parameters and $H\left(U\right)$ is the hop count from candidate upstream U to a base station. The weights $w_{1}$ and $w_{2}$ are applied after candidate upstreams have passed the basic feasibility constraints, including the $R_{c2}$ communication-radius constraint, finite route validity, hop-increase constraint, and loop-free validation. Therefore, the score is not used as the sole feasibility criterion. In this study, $w_{1}$ emphasizes pairwise link stability, whereas $w_{2}$ penalizes excessive route-depth growth. The hop-count term is therefore interpreted as a route-depth control penalty rather than an independent disqualification rule.

To prevent excessive route elongation after repair or optimization, this study also imposes a hop-increase constraint. If the original route hop count of C is denoted by $H_{old}$, the new candidate must satisfy

(8) $$H\left(U\right)+1\leq H_{old}+\Delta H_{MAX},$$

The hop-increase constraint is applied only when the current CH has a finite previous valid hop count. If a finite $H_{old}$ is available, the repaired route must satisfy Equation (8). If the current CH is already disconnected and no finite previous valid hop count is available, the relative hop-increase constraint is not applied during the initial repair attempt. In this case, the candidate upstream must still satisfy the basic route-validity requirements: the candidate must have a finite valid path to a base station, the CH-to-candidate distance must satisfy $d(C,U)\leq R_{c2}$, and the resulting upstream chain must pass loop-free validation. Therefore, the absence of Hold does not block CH-HELP repair, but upstream path validity remains mandatory.

Under the communication-reachability condition $d(C,U)\leq R_{c2}$, the route-validity condition, and the applicable hop constraint in Equation (8), the candidate that maximizes Equation (7) is selected as the best upstream so as to balance link stability and end-to-end route efficiency.

#### 3.3.6. Handover Trigger Condition

Based on the energy and stability definitions above, this study further formulates the event condition under which a cluster head C should initiate handover at time t:

(9) $$T_{handover}\left(C,t\right)=\left\{\begin{array}{ll} 1, & \;\left[E\left(C\right)<E_{TRIGGER}\right]\wedge \left[\exists n\in M\left(C,t\right):E\left(n\right)\geq E\left(C\right)+\Delta E\right],\\ 1, & S_{self}\left(C,t\right)<S_{THRESHOLD},\\ 0, & \mathrm{otherwise}. \end{array}\right.$$

The first condition corresponds to energy-triggered handover. It is activated only when the current CH enters a low-energy risk state and at least one member node has a sufficient residual-energy advantage over the current CH. The second condition corresponds to stability-triggered handover. It is activated when the current CH becomes self-unstable according to the self-stability threshold. The trigger condition determines whether handover evaluation is needed, whereas the final successor is selected through the local backoff competition described in Section 4.

### 3.4. Design Objectives

The proposed mechanism is designed with three practical objectives under continuous drifting: (1) to reduce maintenance gaps by enabling sub-step-level localized recovery; (2) to limit unnecessary network-wide reorganization by prioritizing event-triggered local repair; and (3) to constrain long-term route degradation through topology-quality monitoring and conditional global correction. In this paper, “event-driven” therefore refers to finer maintenance granularity within the adopted discrete-time framework, rather than continuous-time zero-latency reaction.

## 4. Event-Driven Self-Healing Routing Maintenance Mechanism

### 4.1. Event-Driven Cluster-Head Handover

To prevent cluster failure and backbone disruption caused by CH energy depletion or unstable drifting behavior, this study designs an event-driven CH handover mechanism. Unlike traditional periodic re-election schemes, the proposed mechanism is triggered locally only when specific risk conditions arise, thereby reducing reliance on fixed-schedule network-wide maintenance while improving handover responsiveness.

#### 4.1.1. Energy-Triggered Handover

To prevent a cluster head from exhausting its energy and failing, which would disconnect both the cluster and the backbone network, this study designs a two-gate detection mechanism so that the handover decision reflects both low-energy risk and the practical benefit of role transfer. The mechanism consists of the following two conditions.

**Gate A: Residual-energy warning.** The system first checks whether the residual energy E(C) of the current CH falls below the predefined trigger threshold $E_{TRIGGER}$. If

(10) $$E(C)<E_{TRIGGER},$$

the CH is deemed to have entered a low-energy risk zone, and the successor-eligibility checking procedure is initiated.

**Gate B: Relative energy advantage.** Satisfying Gate A alone does not immediately execute handover. The system must further determine whether a candidate successor exists within the cluster that can offer substantial improvement. The old CH searches only among its current cluster members M(C,t) for a candidate successor n, and checks whether its residual energy E(n) satisfies

(11) $$E\left(n\right)\geq \;E(C)+\Delta E,$$

Here, C denotes the current CH, n denotes a candidate successor, and ΔE is the energy-difference threshold. Only when a candidate node has sufficiently higher energy than the current CH is the handover considered to provide a meaningful benefit in redistributing the cluster-head role, and the handover process is then formally activated.

Here, M(C,t) denotes the set of ordinary member nodes currently associated with the current CH C. Neighboring nodes outside the current cluster are not considered as energy-triggered handover successors.

#### 4.1.2. Stability-Triggered Handover

In addition to energy depletion, drifting and relative motion in marine environments also constitute important causes of link breakage. Even when a CH still has sufficient residual energy, excessively rapid drifting or poor local link stability may still weaken the cluster structure and eventually cause failure of the backbone route. Accordingly, this study introduces a self-stability indicator $S_{self}$ to capture the CH-level drift intensity of an individual node and to identify whether the current CH is becoming unsuitable for the CH role.

A stability threshold $S_{THRESHOLD}$ is defined. The system continuously monitors the $S_{self}$ value of each CH, and when

$$S_{self}(C,t)<S_{THRESHOLD},$$

the CH is regarded as having entered an unstable state. This condition does not directly execute handover. Instead, it initiates a stability-triggered handover evaluation.

Unlike energy-triggered handover, the emphasis of stability-triggered handover is preventive maintenance. When a CH has not yet become energy-critical but has already exhibited high-risk drifting behavior, the system may proactively evaluate CH handover to reduce the risk of later backbone disruption. The final successor is then determined through the local backoff competition described in Section 4.1.3.

#### 4.1.3. Handover Procedure and Route Inheritance

When the energy-triggered condition or the stability-triggered condition is satisfied, the old CH initiates a handover evaluation process. The actual CH role transfer is executed only when a qualified successor is selected through the local backoff competition. To preserve backbone continuity while minimizing control cost, this study proposes a locally competitive handover and route-inheritance procedure consisting of the following main steps.

To ensure dimensional consistency, the predicted drift term is normalized by the reference velocity scale before being combined with the other priority terms. Let $\eta_{U}$ denote the normalized predicted drift term, defined as

(12) $${\;\eta }_{U}=\frac{\left\|U_{0}\right\|}{V_{ref}}\;\;\;\;\;\;$$

Here, $U_{0}$ is the predicted drift velocity vector of the candidate node, and $V_{ref}$ is the reference speed scale used in the self-stability definition. This normalized form makes the predicted-drift term dimensionless before it is combined with the other backoff-priority terms.

**Handover announcement.** The old CH broadcasts a handover-evaluation message within the cluster to inform member nodes that local successor competition has been initiated.

**Backoff competition.** Once handover has been triggered, the final successor is not determined directly by the trigger condition itself. Instead, candidate nodes compute their backoff times according to execution history, forwarding burden, the normalized predicted drift term, and a random perturbation term. The backoff time is defined as

(13) $$\begin{array}{rr} \mathrm{backoff}\;\mathrm{time}= & T_{1}\frac{R_{execute}}{R_{current}}+T_{2}\frac{\sum_{k=1}^{R_{current}}\left(\frac{N_{pass-through}\left(k-1\right)}{N_{cluster}\left(k-1\right)+N_{pass-through}\left(k-1\right)}\right)}{R_{execute}+1}\\ & +T_{3}\eta_{U}+T_{4}\;(1-S_{up})+T_{5}\;\xi . \end{array}$$

Here,

1. $R_{execute}$ denotes the accumulated time during which the node has participated in network operation;
2. $R_{current}$ denotes the total number of elapsed simulation hours at the time when backoff competition is evaluated. The backoff rule is evaluated only after the simulation enters the first valid elapsed-time state, so $R_{current}$ is not zero during actual backoff calculation;
3. $N_{pass-through}\left(k\right)$ denotes the number of forwarding packets carried by the node in the k-th time unit; if the node is not a CH during that period or if forwarding history is insufficient at initialization, this value is treated as 0;
4. $N_{cluster}\left(k\right)$ denotes the cluster size associated with the node in the *k*-th time unit;
5. $\eta_{U}\;$ denotes the normalized predicted drift term computed from the same locally corrected current–wind weighted velocity field used in the node-motion update.
6. $T_{1},T_{2},T_{3},T_{4},T_{5}$ are weighting parameters corresponding to execution history, forwarding burden, the normalized predicted drift term, upstream-feasibility adjustment, and random perturbation, respectively.
7. ξ ~ Unif(0,1) is a bounded random perturbation term used for tie-breaking among candidates with similar backoff priorities.

At initialization, if execution or forwarding history is unavailable, the corresponding history-dependent terms are initialized to 0. The $R_{execute}+1$ denominator prevents division by zero for candidates with no prior execution record and smooths early-stage backoff calculation when execution history is still limited.

The backoff formulation combines execution history, forwarding burden, the normalized predicted drift term, upstream feasibility, and bounded random tie-breaking. Residual energy is handled in the handover-trigger and successor-eligibility conditions rather than being introduced again as a continuous ranking term in Equation (13).

A shorter backoff time corresponds to a higher priority in the local handover competition and therefore a higher chance of becoming the new CH.

The trigger conditions decide whether handover evaluation starts, whereas the backoff rule determines which qualified candidate becomes the successor.

**New-CH selection.** If a qualified candidate successfully wins the local backoff competition, the candidate whose backoff timer expires first is elected as the new CH and broadcasts an announcement message. Upon receiving this message, all other candidates terminate their competition and update their local association state.

**Route inheritance.** Once a qualified successor has been selected, the new CH preferentially inherits the upstream node ID and hop level of the old CH to reduce the cost of backbone reconstruction.

The overall event-driven CH handover workflow, including trigger evaluation, local competition, successor announcement, and route inheritance with upstream validation, is summarized in Algorithm 1.
**Algorithm 1.** Event-Driven CH HandoverFor each current CH C in the wireless sensor network
    Check whether the current upstream of C remains valid.
    If the current route of C is disconnected, invoke CH-HELP(C) to repair the route.
    Set handoverFlag = false.
    If *E*(*C*) < *E_trigger_* and there exists a member node *n*
*ϵ*
*M*(*C*,*t*) such that *E*(*n*) ≥ *E*(*C*) + ∆*E*, set handoverFlag = true and mark the handover as energy-triggered.
    If *S_self_*(*C*,*t*) < *S_threshold_*, set handoverFlag = true and mark the handover as stability-triggered.
    If handoverFlag = true, broadcast a “Handover Evaluation Start” packet to cluster members.
          Candidate member nodes enter local backoff competition according to the backoff rule.
        Compute each candidate backoff according to the priority rule in Equation (13).
          If no candidate responds or no candidate backoff timer expires within the allowed competition window, cancel the role-transfer attempt and keep C as the current CH.
        Otherwise, if candidate m expires first and no CH_DECLARE packet has been received, m announces itself as the new CH.
        m inherits the old CH’s upstream information.
        Validate the inherited upstream within *T_inherit_*
        If the inherited upstream is invalid, set *S_up_*(*m*) = 0 and invoke CH-HELP(m).
        End if
    End if
End for
End Algorithm


However, inheriting the upstream information of the old CH does not guarantee that the new CH still has a valid upstream path. Therefore, after route inheritance, the new CH sends a local route-validation message to the inherited upstream and waits for a reply within a short time window $T_{inherit}$. If the reply confirms valid uplink capability, the inherited route is retained and $S_{up}$ remains 1; otherwise, the inherited route is regarded as invalid, $S_{up}$ is set to 0, and the CH immediately enters the CH-HELP procedure.

Some member nodes may temporarily lose association during handover. In such cases, they subsequently enter the node-recovery procedure to re-establish a valid cluster association.

During normal operation, each CH maintains only lightweight local routing state, including its current upstream ID, hop level, and upstream-validity state. Ordinary data packets do not carry a complete end-to-end upstream chain, and CHs do not continuously synchronize complete routing tables.

However, this local-state design does not imply that path-level validation is never performed. Additional route-validation and loop-avoidance checks are activated only on demand during route inheritance, CH-HELP, and route-repair procedures. When a candidate upstream is evaluated, the candidate route is temporarily validated to determine whether it remains reachable and loop-free. Therefore, the proposed mechanism uses lightweight next-hop operation during normal reporting and event-triggered upstream-chain validation only when repair decisions require it.

After route inheritance, the new CH does not retain the inherited upstream blindly. Instead, the inherited route is validated through bounded upstream-chain validation. The validation checks whether each upstream hop satisfies the $R_{c2}$ reachability condition, whether each upstream CH is alive, whether the chain reaches a base station, and whether the chain forms a loop. If validation fails, the inherited route is marked invalid and CH-HELP is activated.

### 4.2. Dual-Layer Self-Healing Recovery Mechanism

Because ocean-current drifting is continuous and uncertain, even preventive handover cannot completely avoid disconnection events. To ensure that the network can rapidly restore reachability after link breakage, this study proposes a layered recovery strategy with dedicated mechanisms for backbone repair and ordinary member-node reattachment.

Backbone recovery is organized around the upstream-validity state of each CH. When an upstream CH restores a valid uplink, its downstream child CHs may also recover through subsequent route-validation updates without immediately restarting HELP, thereby reducing redundant requests and repair oscillation.

#### 4.2.1. CH-HELP Mechanism

Under the proposed mechanism, a CH determines uplink failure from locally observable protocol events rather than from continuously synchronized global route knowledge. When route-validation updates or upstream responses can no longer be maintained within the allowed monitoring and retransmission conditions, the CH sets $S_{up}$ from 1 to 0 and triggers CH-HELP to reconstruct an uplink path without destroying the current cluster structure.

CH-HELP should be understood as a localized upstream-restoration procedure within the proposed maintenance framework. Its role is to recover backbone reachability under local fragmentation while respecting upstream-validity, hop-growth, and loop-avoidance constraints.

**(1) Direct-BS check.** A failed CH first checks whether it can establish a direct connection to any base station. If, based on local control signaling with the base station, short-term link-quality estimation, and stability evaluation, the CH determines that a stable direct BS connection is feasible, then the system preferentially sets the base station as its upstream, resets its hop level to 1, and restores $S_{up}$ to 1. This avoids unnecessary multi-hop recovery and relay burden, while also shortening the recovered route.

Direct-BS feasibility is determined from local confirmation signals rather than global geometric knowledge.

**(2) Best-upstream search.** If the CH cannot directly connect to a base station, it broadcasts a CH-HELP request to nearby CHs within $R_{c2}$. A candidate CH U responds only if $S_{up}(U)=1$ has already been confirmed in the current BS-outward validation order, the requesting CH-to-candidate distance satisfies $d(C,U)\leq R_{c2}$, and the bounded validation procedure in Section 4.3 confirms that selecting U does not create a routing loop involving the requesting CH C. CHs that have already lost upstream validity do not participate in the response.

The failed CH then selects the best upstream according to the scoring function defined in Section 3.3.5:

$$Score\left(C\to U\right)=w_{1}S_{link}\left(C,U\right)-w_{2}H(U)$$

Candidate upstreams in CH-HELP must satisfy valid-uplink, communication-radius, hop-increase, and loop-avoidance constraints. Loop avoidance is not checked only by a single upstream ID comparison. Instead, the upstream chain of each candidate is traversed using a visited set. If the traversal revisits the requesting CH or any previously visited CH, the candidate upstream is marked invalid.

Route validation is propagated in a BS-outward manner. CHs closer to the base station first confirm their uplink validity, and only CHs with confirmed upstream validity are used as feasible upstream candidates by downstream CHs. Therefore, a downstream CH does not simply attach to a geometrically reachable CH; the candidate upstream must have passed upstream-validity confirmation from the upper layer.

Once CH-HELP succeeds, the recovered CH restores its upstream state, updates its hop level, and propagates route-recovery notifications to its direct downstream child CHs. This allows dependent downstream CHs to update or revalidate their states without immediately launching independent HELP procedures.

If CH-HELP fails, the CH remains temporarily unreachable with $S_{up}=0$, and the failure may subsequently propagate downward along backbone dependency.

The detailed CH-HELP backbone repair procedure, from direct-BS checking to best-upstream reselection and downstream validity propagation, is summarized in Algorithm 2.
**Algorithm 2.** Cluster Head Help (CH-HELP)When CH node *n* detects a disconnection in its path to BS
    Record the current state of *n* as “Disconnected”
    Set *S*_up_(*n*) = 0
    If a direct BS connection is feasible under the *R*_*c*2_ based local confirmation condition
        Set the BS as the upstream of *n*
        Set *n*.Hop = 1
        Set *S*_up_(*n*) = 1
        Update the state of *n* to “Reconnected”
    Else
        CH *n* broadcasts a CH-HELP Request packet with radius *R_c_*2
        For each alive CH node *m* within communication range
            If *S*_up_(*m*) = 1 has been confirmed in the current BS-outward validation order AND the bounded validation in Section 4.3 confirms that the upstream chain of *m* does not contain *n*
                Calculate Score(*n*,*m*) using Equation (7)
                Reply with a control packet containing the score to node *n*
            End if
        End for
        If node *n* receives one or more reply packets
            Select *best_m_* with the highest score as the new upstream
            Update upstream ID of *n* to *best_m_*
            Update hop level: *n*.Hop = *best_m_*.Hop + 1
            Set *S*_up_(*n*) = 1
            Propagate the route-recovery update to direct downstream child CHs
            Update the state of *n* to “Reconnected”
        Else
            Maintain the state of *n* as “Disconnected”
            Keep *S*_up_(*n*) = 0
        End if
    End if
End Algorithm



#### 4.2.2. Node-HELP Mechanism

When an ordinary member node detects that it has lost connection to its associated CH, the Node-HELP mechanism is triggered. Unlike CH-HELP, which focuses on backbone restoration, Node-HELP aims to assist isolated member nodes in reattaching to a cluster that still has a stable route to a base station.

The recovery process proceeds as follows.

**Isolation broadcast.** The disconnected node broadcasts a HELP message within the intra-cluster communication radius $R_{c1}$ to declare that it has lost cluster association.

**Candidate filtering.** A neighboring CH that receives the HELP request replies only if it satisfies the required condition. Specifically, the CH must still maintain valid uplink capability, that is, its current $S_{up}=1$. Otherwise, even if the member node successfully joins that CH, end-to-end data transmission cannot be completed. This step therefore prevents a node from rejoining a cluster that is already disconnected or unstable.

**Stability-based selection.** After collecting all replies, the isolated node computes the link stability between itself and each candidate CH, selects the most stable one, and updates its CH ID and membership record accordingly. Unlike CH-HELP, whose objective is backbone recovery, Node-HELP mainly focuses on re-establishing edge access. Therefore, it adopts a stability-priority strategy and does not additionally introduce hop penalty, thereby reducing the decision complexity of end-node reattachment.

The Node-HELP member-node reattachment procedure, including HELP broadcasting, candidate filtering, and stability-based CH selection, is summarized in Algorithm 3.
**Algorithm 3.** Node Help (Node-HELP)When node n detects that its connection to the CH is broken or its CH cannot reach the BS
     Record the current state of n as “Disconnected”
     Increment Node-HELP call count and cumulative statistics
     Node n broadcasts a Node-HELP Request packet with radius *R*_*c*1_
     For each alive CH node ch within communication range
            If (ch has a valid route to BS AND ch.Upstream_ID ≠ −1)
                  ch replies with a control packet to node n
                  Calculate link stability: Score = LinkStability(n, ch)
            End if
     End for
     If node n receives one or more replies from candidate CHs
            Select best_CH with the highest stability score
            If n was previously associated with an old CH
                  Remove n from the old CH’s member list
            End if
            Update n.CH_ID = best_CH.ID and set n.is_member = True
            Add n to the best_CH member list
            Update the state of n to “Reconnected”
     Else
            Maintain the state of n as “Disconnected” and set n.CH_ID = 0
     End if
End Algorithm



### 4.3. Bounded Upstream-Chain Validation for Routing Loop Prevention

To prevent routing loops under dynamic topology changes, this study adopts bounded upstream-chain validation during route inheritance, CH-HELP, and upstream reselection. The validation procedure traverses the upstream chain from the requesting CH toward a base station. A visited set is maintained during traversal to detect loops. A candidate upstream is marked invalid if the traversal revisits a CH already stored in the visited set, if any backbone hop exceeds $R_{c2}$, if an upstream CH is not alive, if the upstream is undefined, or if the number of upstream-chain traversal steps reaches $D_{max}$ before a base station is found. In this study, $D_{max}=N_{node}+5$, where $N_{node}$ denotes the total number of simulated sensor nodes. The additional constant is used only as a safety buffer to prevent unbounded traversal; therefore, $D_{max}$ is not a routing-hop design parameter. A candidate route is marked valid only when the traversal reaches a base station through hops satisfying the $R_{c2}$ reachability constraint.

This mechanism is activated only when a route-repair or route-inheritance decision requires path-level validation. During normal reporting, each CH maintains only lightweight local routing state, including its current upstream ID, hop level, and upstream-validity state. Therefore, the proposed design does not require continuously synchronized global route knowledge during ordinary data forwarding.

After successful validation, the repaired CH updates its hop level according to the selected upstream. The repaired CH then sends a local route-validity update to its direct downstream child CHs. Each downstream CH revalidates its immediate upstream state and updates its hop level according to the new upstream hop value. If the downstream CH remains valid, it further propagates the update to its own downstream child CHs. If validation fails, the downstream CH sets $S_{up}$= 0 and enters the corresponding repair procedure. This top-down update prevents downstream CHs from retaining stale hop levels after an upstream repair.

Algorithm 4 represents the simulator-level abstraction of this on-demand validation process. In the reported simulator, bounded validation is implemented by following stored upstream identifiers. This implementation is used to emulate the outcome of a hop-limited validation exchange and should not be interpreted as a continuously available global topology table or as ordinary data packets carrying complete end-to-end paths.
**Algorithm 4.** On-Demand Route Validation and Loop Avoidance
Function RecomputeRoute(Node C)
    If C can directly reach the base station within range *R*_*c*2_
       Set C.Upstream = BS
       Set C.HOP level = 1
       Set *S*_up_(*C*) = 1
       Propagate local route-update information to direct downstream child CHs
       Exit
    Else
       C broadcasts a Route-Request using range *R*_*c*2_
       Initialize CandidateSet = empty
       For each candidate node *U* within *R*_*c*2_
           If *U* is alive
              Initialize CurrentUpstream = *U*
              Initialize VisitedSet = empty
              Initialize Steps = 0
              Initialize IsValid = false
              While Steps < *D*_max_
                 If CurrentUpstream = BS
                     Set IsValid = true
                     Break
                 End if
                 If CurrentUpstream is undefined OR CurrentUpstream is not alive
                     Mark *U* as invalid
                     Break
                 End if
                 If CurrentUpstream is already in VisitedSet
                     Loop detected; mark *U* as invalid
                     Break
                 End if
                 Add CurrentUpstream to VisitedSet
                 Let NextUpstream = CurrentUpstream.Upstream
                 If NextUpstream is undefined
                     Mark *U* as invalid
                     Break
                 End if
                 If distance(CurrentUpstream, NextUpstream) > *R*_*c*2_
                     Mark *U* as invalid
                     Break
                 End if
                 Set CurrentUpstream = NextUpstream
                 Set Steps = Steps + 1
             End while
             If Steps reaches *D*_max_ before reaching a base station, mark *U* as invalid.
             If IsValid = true
                 Calculate Score(C, U)
                 Add *U* to CandidateSet
             End if
           End if
         End for
         If CandidateSet is not empty
             Select *U*_best_ with the maximum score
             Set C.Upstream = *U*_best_
             Set C.HOP level = *U*_best_.HOP level + 1
             Set *S*_up_(*C*) = 1
             Propagate local route-update information to direct downstream child CHs
         Else
             Set C.Upstream = undefined
             Set *S*_up_(*C*) = 0
             Recovery failed; mark C as Disconnected
         End if
   End if
End Function


### 4.4. Adaptive Reorganization Mechanism Based on Topology Quality

Although CH-HELP and Node-HELP support localized repair after disconnections occur, long-term drifting may still cause gradual backbone-path elongation, route detouring, and topology mismatch.

To quantify backbone quality, the BS constructs the reachable-CH set $C_{reach}(t)$ from compact reports successfully received from CHs with valid uplink paths at observation instant t. Let $h_{c}(t)$ denote the hop count of CH c at time t, and let $H_{tail}$ denote the long-hop threshold. Then the tail ratio of long backbone paths is defined as

(14) $$\rho_{tail}\left(t\right)=\frac{\left|\left\{c\in C_{reach}\left(t\right)|h_{c}\left(t\right)\geq H_{tail}\right\}\right|}{\left|C_{reach}\left(t\right)\right|},$$

When $|C_{reach}\left(t\right)|=0$, no reachable CH report is available at the BS, and the hop-depth distribution of reachable CHs cannot be evaluated. In this case, $\rho_{tail}(t)$ is numerically set to 0 only to avoid division by zero. This value is not interpreted as a normal topology state. Instead, the corresponding snapshot is treated as a severe backbone reachability failure caused by missing CH reports.

The conditional global reclustering trigger is evaluated at the BS side rather than by every CH through a network-wide scan. Reachable CHs may include compact state summaries, such as CH ID, hop level, and upstream-validity state, in normal reporting or route-validity messages. The BS computes $\rho_{tail}\left(t\right)$ only from CHs whose summaries successfully reach the BS. CHs that cannot report are treated as reachability failures and are not further classified for hop-depth degradation. When topology summaries or route-validity reports are transmitted as explicit control messages, they are counted in the HighTx component of the control-plane cost index.

To avoid premature triggering of global reorganization due to a single transient fluctuation or temporary local anomaly, this study adopts a persistence-based triggering condition. Specifically, the system concludes that backbone-quality degradation has entered a sustained state only when the $\rho_{tail}\left(t\right)$ at both the previous observation instant and the current observation instant exceeds the predefined threshold $\theta_{tail}$:

(15) $$\rho_{tail}\left(t-1\right)\geq \theta_{tail}\;\mathrm{and}\;\rho_{tail}\left(t\right)\geq \theta_{tail},$$

The proposed global reclustering mechanism serves as a low-frequency corrective measure rather than routine periodic maintenance. A reclustering request is accepted only when the persistence condition in Equation (15) is satisfied and the minimum reclustering interval is met. The value of $\rho_{tail}(t)$ is recorded at every observation after the BS receives the available CH reports, regardless of whether the minimum reclustering interval has been satisfied. Therefore, Equation (15) is used only to detect sustained backbone degradation, whereas $T_{recluster\_min}$ is used only as an execution-frequency constraint for global reclustering. If the persistence condition is satisfied but the minimum interval has not elapsed, global reclustering is not executed at that observation, but the current $\rho_{tail}(t)$ value is still stored for the next persistence evaluation. To avoid control conflict, the request is deferred until ongoing local control procedures finish and the system reaches the next global-maintenance entry point. During global reorganization, clustering and backbone construction are rebuilt to realign the topology with the current node distribution. The procedure is summarized in Algorithm 5.
**Algorithm 5.** Conditional Global Re-clustering with Persistent Tail-Hop DetectionFunction ConditionalGlobalReclustering(round *t*)
    Construct *C*_reach_(*t*) from CHs whose route/status reports successfully reach the BS
    If ∣*C*_reach_(*t*)∣ = 0
       Set *ρ*_tail_(*t*) = 0 for numerical safety
       Mark this observation as a severe backbone reachability failure
       Store *ρ*_tail_(*t*) for persistence evaluation
       Maintain the current topology
       Exit
    End if
    Calculate *ρ*_tail_(*t*) from *C*_reach_(*t*)
    Store *ρ*_tail_(*t*) for persistence evaluation
    Set persistenceFlag = false
    If *ρ*_tail_(*t* − 1) ≥ *θ*_tail_ AND *ρ*_tail_(*t*) ≥ *θ*_tail_
       Set persistenceFlag = true
    End if
    If persistenceFlag = false
       Maintain the current topology
       Exit
    End if
    If *t* − lastReclusterRound < *T*_recluster_min_
       Do not execute global re-clustering at this observation
       Maintain the current topology and keep the stored *ρ*_tail_(*t*)
       Exit
    End if
    If ongoing local repair, handover, or CH-HELP control procedures are active
       Defer the global re-clustering request until the next global-maintenance entry point
       Exit
    End if
Trigger global re-clustering
Reset active node roles, including CH and member states.
Clear routing tables and hop levels.
Execute CH selection to select new cluster heads.
Execute backbone route setup to rebuild the backbone routing topology.
Update lastReclusterRound = t.
End Function



### 4.5. Complexity Analysis

To provide a formal complexity characterization of the proposed maintenance framework, this subsection summarizes the computational and control-message complexity of the main maintenance components. Let N denote the total number of sensor nodes, and let K denote the number of cluster heads. For a given cluster head C, $m_{C}=|M\left(C\right)|$ denotes the number of member nodes associated with C, and $d_{CH}(C)$ denotes the number of neighboring CHs within the backbone communication radius $R_{c2}$. For an ordinary node i, $d_{node}(i)$ denotes the number of candidate CHs reachable by i. The parameter $D_{max}$ denotes the maximum depth allowed in bounded upstream-chain validation.

The local maintenance operations are bounded by the local cluster size, neighboring-CH degree, candidate-CH degree, or the maximum upstream-validation depth. Therefore, CH handover, route inheritance validation, CH-HELP, and Node-HELP do not require network-wide recomputation during ordinary repair events.

The only network-wide component is conditional global reclustering. Its worst-case computational complexity is $O(NK+K^{2})$, where O(NK) corresponds to node-to-CH association evaluation and $O(K^{2})$ corresponds to CH-to-CH backbone reconstruction in a dense candidate setting. However, this operation is not executed periodically in every round; it is triggered only when the $\rho_{tail}(t)$ based degradation condition persists and the minimum reclustering interval is satisfied. Thus, the proposed framework shifts most maintenance cost from repeated global reconstruction to localized event-driven repair. The computational and control-message complexity of the main maintenance components is summarized in Table 3.

## 5. Results and Discussion

The detailed deployment-boundary coordinates, simulation-configuration record, and run-level numerical result tables are provided in the Supplementary File S1 package. The S1 package contains a reproducibility PDF document and an Excel workbook with the run-level numerical and raw-result tables.

The drift update in this study is implemented as a current-dominant flow–wind weighted drift field. The ocean-current component is used as the primary drift component, while the wind-field component is incorporated as a secondary drift-adjustment term through the weighting factor f. Wave- and tide-induced effects are not explicitly modeled as separate physical forcing terms in the present topology-level simulation.

The combined drift velocity is represented as $u_{B}=f\;u_{current}+(1-f){\;u}_{wind}$ and $v_{B}=f\;v_{current}+\left(1-f\right)v_{wind}$, where f=0.97 assigns dominant weight to the ocean-current component. This setting reflects the current-dominant drift assumption adopted in this simulation and prevents the wind term from overwhelming the current-driven trajectory.

For a fair comparison with DARCR, the study area is located in the Taiwan Strait and is modeled as a 100 × 100 km sea region, corresponding to a total area of 10,000 km^2^. Two fixed base stations are positioned at the trisection points along one boundary of the monitored area. Node drifting trajectories are generated using a current-dominant flow–wind drift field constructed from Copernicus Marine Service environmental data. The detailed simulation parameters are listed in Table 4.

The environmental drift inputs used in this study consist of ocean-current and wind-field data over the Taiwan Strait region. The ocean-current input was obtained from the Copernicus Marine Service product GLOBAL_ANALYSISFORECAST_PHY_001_024, Global Ocean Physics Analysis and Forecast. The wind-field input was obtained from the Copernicus Marine sea-surface wind product Global Ocean Hourly Sea Surface Wind and Stress from Scatterometer and Model. The processed ocean-current input has an approximately 1/12° × 1/12° spatial grid, whereas the processed wind-field input has a 0.125° × 0.125° spatial grid. Both datasets are organized at hourly temporal resolution and converted into u/v components before being used in the simulator. During simulation, each node is matched to the nearest available ocean-current and wind-field grid points at the corresponding hourly timestamp, followed by covariance-based local correction using nearby grid points within a predefined distance threshold. Within each hourly environmental update, node positions are advanced at six 10 min sub-steps using the updated weighted drift velocity field.

Unless otherwise specified, the primary simulation results presented in this study are the averages derived from 10 independent stochastic simulation runs under the same fixed environmental forcing data. The ocean-current and wind-field inputs are fixed across runs, whereas node initialization and stochastic simulation processes vary across independent runs. The purpose of employing multiple independent simulations is to mitigate the impact of individual topology initializations and stochastic fluctuations on the results. This approach ensures that the comparative findings more accurately reflect the overall performance of the methods within a drifting environment.

### 5.1. Ablation Study of Key Maintenance Components

Before comparing the proposed method with external baselines, this subsection first examines the individual contributions of its key maintenance components. Specifically, CH-HELP, Node-HELP, and conditional global reclustering are removed one at a time to clarify how each component affects connectivity preservation and member reattachment under continuous drift.

In addition to the CH-level disconnection rate used in the main DARCR comparison, this ablation study further introduces an overall disconnection rate, defined as the snapshot-based end-to-end unreachability ratio over all nodes. The reason is that Node-HELP primarily affects member-node reattachment rather than backbone CH reachability. Therefore, an overall node-level metric is more suitable for revealing its contribution.

**Figure 1:**
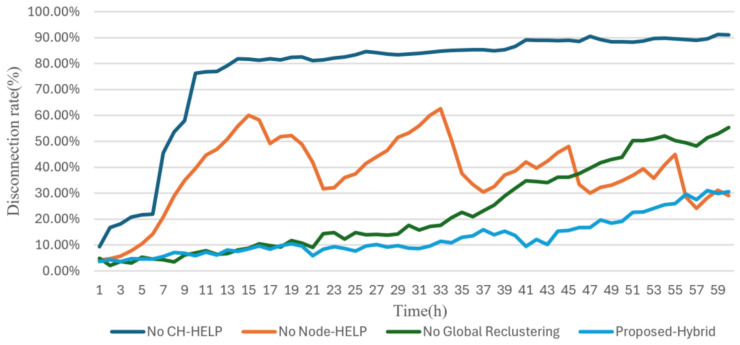
Comparison of overall disconnection rates under ablation settings.

Figure 1 compares the performance of the full Proposed-Hybrid method with three ablation versions: one without CH-HELP, one without Node-HELP, and one without conditional global reclustering. The results demonstrate that the full method achieves the lowest mean overall disconnection rate at 12.88%. Removing the conditional global reclustering increases the rate to 23.15%, with a persistent deteriorating trend in the later stages. The absence of Node-HELP further raises the rate to 37.73%. Most significantly, without CH-HELP, the disconnection rate surges to 76.91% and remains at this high level for a prolonged period. Overall, these findings indicate that CH-HELP, Node-HELP, and conditional global reclustering play complementary roles: CH-HELP is most critical for maintaining backbone reachability, Node-HELP primarily facilitates the reattachment of regular nodes, and conditional global reclustering serves to suppress late-stage topology degradation caused by long-term drift.

Notably, the differences between the curves are evident not only in their mean values but also in their temporal evolution patterns. In the version without Node-HELP, although the curve is significantly higher than that of the full method, it does not remain at a monotonically deteriorating high level. This is primarily because conditional global reclustering can still indirectly restore the reachability of some regular nodes through low-frequency condition-triggered topology correction, resulting in noticeable fluctuations. In contrast, when CH-HELP is removed, the local repair capability at the backbone level is lost. Consequently, not only are CHs unable to restore their uplinks locally, but the triggering of conditional global reclustering—which relies on topology quality information reported by still-connected CHs—also becomes impaired. Furthermore, without conditional global reclustering, although CH-HELP and Node-HELP can still handle most localized anomalies, the lack of a global correction mechanism for long-term topology aging results in a gradual rise in disconnections during the later stages. These results collectively demonstrate that the superior performance of the full method is not achieved by any single module, but through the synergistic effects of all three mechanisms in backbone maintenance, member reattachment, and long-term topological correction.

### 5.2. Comparison with DARCR

For fairness, all baseline methods were evaluated under the same simulation environment, including the same monitored area, node-number setting, base-station placement, current-dominant flow–wind forcing data, simulation duration, communication radii, and random-deployment procedure.

This subsection compares DARCR and the full Proposed-Hybrid method under the same drifting environment and parameter settings. The reported CH-disconnection rate and hop-based delay proxy are based on averages over 10 independent simulation runs. Here, Proposed-Hybrid denotes the full proposed framework, including handover, CH-HELP, Node-HELP, route inheritance, loop prevention, and conditional global reclustering. The local-only configuration without conditional global reclustering, denoted as Proposed-Local, is analyzed separately in Section 5.3.4 to isolate the contribution of the global corrective layer. Unless otherwise specified, “Proposed” in the comparison figures denotes Proposed-Hybrid.

The DARCR results reported in this subsection were obtained by re-running DARCR under the same N = 1300 configuration, communication-radius assumptions, base-station placement, current-dominant flow–wind forcing, and simulation duration as the proposed method.

#### 5.2.1. Hop-Based Delay Proxy

Following the hop-based delay evaluation used in DARCR, this study adopts a 1 s per hop delay proxy to compare end-to-end transmission timeliness under different topology-maintenance strategies. This delay metric is used as a relative routing-depth indicator rather than a physical-layer latency prediction, so that the comparison is not dominated by implementation-dependent factors such as MAC contention, queueing delay, retransmission, synchronization overhead, interference, or sea-state-dependent link variation. The CH-to-BS hop count H is therefore used as an approximation of end-to-end delay:

(16) $$D_{e2e}\approx H\times 1\;s,$$

Therefore, the results should be interpreted as a hop-depth-based timeliness proxy rather than a direct measurement of actual end-to-end latency.

**Figure 2:**
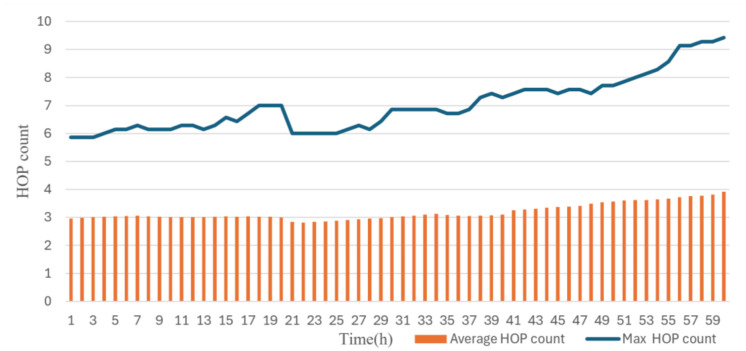
Average and maximum hourly hop count.

Figure 2 shows the hourly variations in the average and maximum hop counts during the simulation. The bars in the graph represent the average hop count per hour. The figure indicates that the average hop count is approximately 3 in the early stage of network operation. As drifting gradually stretches the topology and increases the need for route detouring, the average hop count slowly rises to approximately 4, implying that, under the hop-based approximation, the average reporting timeliness proxy remains within about 4 s.

The line in the figure represents the maximum number of hops, which reflects the worst-case route length and tail-end risk. Over the 60 h simulation period, the maximum hop count remains below 10, indicating that under the same approximation, the proxy-level upper bound for farthest-node reporting is about 10 s. The above simulation analysis of hop count demonstrates that the proposed scheme achieves a transmission efficiency comparable to DARCR.

#### 5.2.2. Disconnection Rates

In this subsection, for consistency with the DARCR baseline, the reported CH disconnection rate is evaluated at the CH level. A CH is regarded as disconnected when its current uplink route to a base station becomes invalid. Therefore, the metric reported here is the snapshot-based CH disconnection ratio at each observation time, rather than the cumulative number of disconnection events defined in Section 3.

This definition is consistent with the use of $S_{up}$ in Section 3 to represent upstream validity. When a CH has $S_{up}=0$, its current upstream is considered invalid, and the resulting unreachable state is also reflected in downstream nodes associated with that CH. The route-validation update, CH-HELP, and Node-HELP mechanisms described in Section 4 correspond to the detection and repair of this unreachable state.

**Figure 3:**
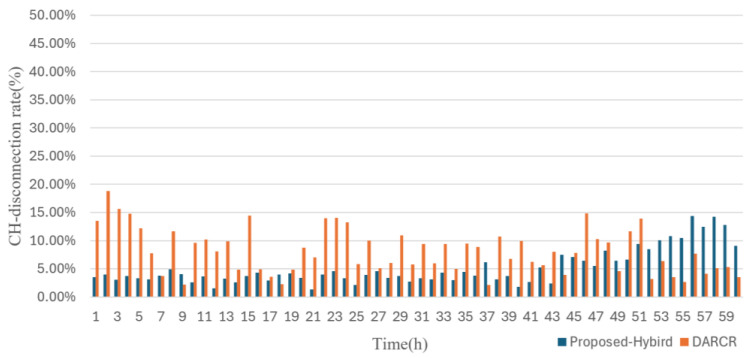
Comparison of CH-disconnection rates between Proposed-Hybrid and DARCR.

Figure 3 compares the evolution of CH-disconnection rate over time for Proposed-Hybrid and DARCR. The results show that Proposed-Hybrid maintains a lower full-period mean CH-disconnection rate under the same drifting environment, indicating that the full event-/condition-triggered maintenance framework improves average CH-level connectivity when event-driven local repair and conditional global reclustering are jointly enabled. However, this full-period advantage should not be interpreted as uniform dominance over DARCR across all late-stage metrics. DARCR relies on fixed-period reorganization, which can periodically refresh the topology and remain competitive in some final-third or peak-disconnection indicators.

According to the experimental statistics averaged over 10 independent simulation runs, the mean CH-disconnection rate of Proposed-Hybrid is 5.15%, whereas DARCR yields 8.15%. This indicates that the full Proposed-Hybrid framework improves CH-level connectivity and reporting reliability under the same environmental conditions. A consolidated statistical summary for DARCR, Proposed-Local, and Proposed-Hybrid, including confidence intervals, is reported in Section 5.3.4.

#### 5.2.3. Direct Statistical Comparison with DARCR

To make the comparison with DARCR explicit, this subsection summarizes the direct statistical comparison between DARCR and Proposed-Hybrid. The comparison includes CH-disconnection metrics, route-depth indicators, and the control-plane cost index, which together correspond to reporting reliability, backbone-route elongation, and maintenance overhead.

The values in Table 5 are computed from run-level summary values over 10 independent simulation runs. For each run, the full-period mean, final-third mean, and late-stage 95th-percentile peak are first calculated independently. The reported mean and 95% confidence interval are then computed across the 10 run-level values, rather than across hourly samples within the same run. Statistical differences between DARCR and Proposed-Hybrid are evaluated using Welch’s two-sample *t*-test.

As shown in Table 5, Proposed-Hybrid significantly reduces the full-period mean CH-disconnection rate from 8.15 ± 0.44% in DARCR to 5.15 ± 0.42% (Welch’s two-sample *t*-test, *p* = $1.43\times 10^{-9}$), indicating better average CH-level connectivity over the full simulation period. However, DARCR remains more competitive in the final-third mean CH-disconnection rate, where DARCR achieves 6.89 ± 0.64% compared with 8.51 ± 1.49% for Proposed-Hybrid (*p* = 0.0437). For the late-stage 95th-percentile peak, the difference between DARCR and Proposed-Hybrid is not statistically significant, with 16.40 ± 0.83% and 17.21 ± 2.61%, respectively (*p* = 0.5178). Therefore, the advantage of Proposed-Hybrid should be interpreted as lower full-period CH disconnection and event-driven local-repair responsiveness, rather than universal dominance over DARCR in every late-stage metric.

Table 6 further reports route-depth and control-cost indicators. Proposed-Hybrid and DARCR show nearly identical mean average hop values, indicating that the proposed event-driven maintenance framework does not increase the average routing depth. Proposed-Hybrid also achieves a lower mean maximum hop than DARCR, suggesting better control of worst-case route elongation. However, this improvement is accompanied by a higher $C_{cp}(256)$, reflecting the additional backbone-level control burden introduced by event-driven repair and conditional global reclustering.

### 5.3. Investigation of Late-Stage Connectivity Degradation

After comparing the full Proposed-Hybrid method with DARCR, this subsection further investigates the internal contribution of the main maintenance components. In particular, Proposed-Local denotes the local-only configuration that includes handover, CH-HELP, and Node-HELP, but excludes the $\rho_{tail}\left(t\right)\;$ based conditional global reclustering. By comparing Proposed-Local with Proposed-Hybrid, this subsection evaluates whether local repair alone is sufficient under prolonged drifting and quantifies the contribution of the global corrective layer. Unless otherwise specified, the disconnection results reported in this subsection are CH-disconnection rates.

#### 5.3.1. Mechanism Validation

**Figure 4:**
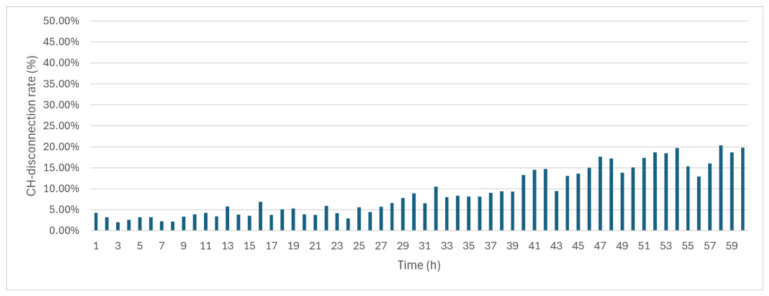
Disconnection-rate evolution.

**Figure 5:**
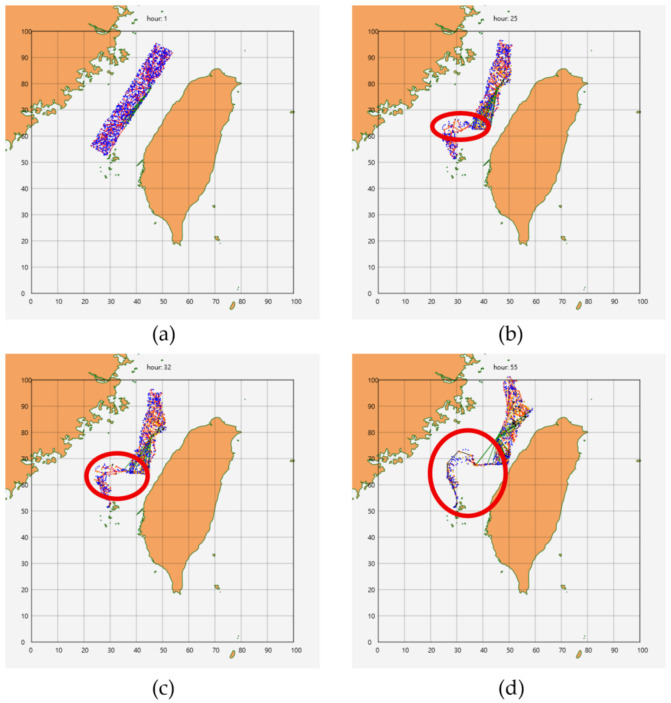
(**a**) Initial backbone. (**b**) Fragile-backbone warning sign before the surge. (**c**) Temporary recovery. (**d**) Late-stage degraded backbone pattern. Blue dots represent member nodes, red dots represent cluster heads (CHs), orange dots indicate CHs undergoing handover, and black dots denote dead nodes. Green links represent direct CH-to-BS connections (Hop 1), red links indicate connected CH-to-CH backbone routes that can still reach the BS, and black links indicate disconnected backbone segments. Red circles highlight representative regions of backbone fragility, temporary recovery, or late-stage degradation discussed in panels (**b**–**d**).

To validate the observed late-stage connectivity degradation, a representative simulation instance is selected for backbone-topology analysis. Figure 4 shows the evolution of the CH-disconnection rate, while Figure 5 presents the corresponding topology snapshots at different stages. The selected case captures typical behavior observed in the simulations, including early-stage variation, temporary recovery, and subsequent degradation.

Initial backbone (Figure 5a): after the initial route construction, the CHs form a relatively well-connected backbone toward the base station, and the overall structure still preserves some degree of redundancy.

Warning signs before the surge (Figure 5b): even while the CH disconnection rate remains low before hour 27, the backbone has already developed a locally fragile bottleneck structure (circled in red), where the reachability of the downstream region appears to rely on only a limited number of connecting links.

Temporary recovery (Figure 5c): by around hour 30–32, the topology shows a partial reconnection of the affected region, consistent with the temporary reduction in the CH disconnection rate.

Late-stage persistent degradation (Figure 5d): near the end of the simulation, the southern region exhibits a less favorable spatial configuration and a more fragile backbone pattern, making it harder for the network to recover a stable and redundant structure. As a result, the CH disconnection rate rises again and remains elevated.

This representative case supports the proposed interpretation: the late-stage high CH disconnection rate is not caused by an isolated random link failure, but by structural fragility resulting from gradual backbone mismatch under prolonged drifting. In such a situation, local HELP alone may be insufficient to reshape the backbone into a structure compatible with the current spatial distribution, and a certain degree of global refresh becomes necessary as a supplementary mechanism.

#### 5.3.2. Role of Global Reclustering

Global reclustering serves as a supplementary safeguard rather than a replacement for local HELP-based repair. It is intended as a low-frequency topology-correction mechanism that is triggered only when persistent backbone-quality deterioration indicates that local repair is no longer sufficient.

In preliminary attempts, two hop-based indicators were considered as potential triggers: average hop count and maximum hop count. Average hop count can reflect the overall hop level of the network, but it is easily diluted by a large number of short-hop nodes, making early-stage backbone degradation difficult to detect. Maximum hop count, although capable of capturing extreme long paths, is overly sensitive to one or a few nodes and may therefore lead to false triggering or difficult calibration. Moreover, a high maximum hop count does not necessarily imply that the overall backbone has degraded. Taken together, neither average hop count nor maximum hop count provides a sufficiently robust trigger threshold for global reclustering.

Accordingly, this study instead adopts the reachable-tail ratio $\rho_{tail}()$, as defined in Section 4.4. The central idea is that when the proportion of long-path CHs among those still reachable to a base station continues to increase, the backbone is gradually deviating from the current spatial distribution of nodes, and local repair may no longer be sufficient to preserve route efficiency. Specifically,$\rho_{tail}(t)$ measures the fraction of CHs in the set $C_{reach}(t)$ whose hop counts satisfy $h_{c}(t)$ ≥ $H_{tail}$, where $C_{reach}(t)$ denotes the set of CHs that still maintain valid multi-hop paths to a base station at time step *t*.

This metric offers three advantages:

1. it is not dominated by a single maximum value;
2. it is not easily diluted by a large number of short-hop nodes; and
3. it has an intuitive interpretation: the higher the proportion of long-hop CHs, the more nodes in the backbone must rely on multi-hop forwarding to remain reachable, and thus the greater the potential risk in route quality, reporting timeliness, and forwarding burden.

Furthermore, to avoid false triggering caused by transient fluctuations, this study adopts a persistence condition: global reclustering is triggered only when $\rho_{tail}\left(t\right)$ remains high continuously, indicating that the backbone degradation has entered a sustained state.

#### 5.3.3. Improvement Results

**Figure 6:**
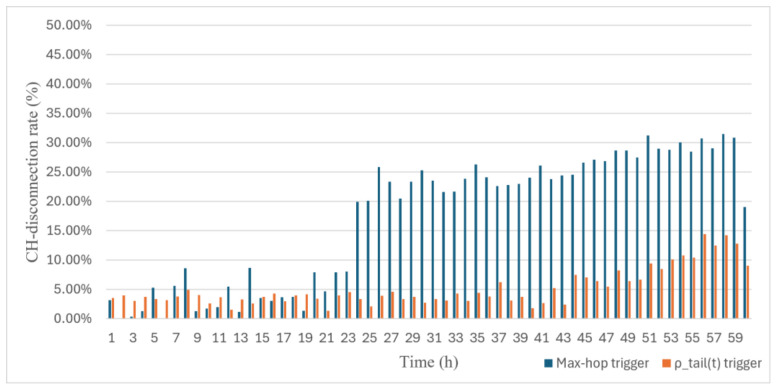
Disconnection-rate results when $\rho_{tail}\left(t\right)$ vs. maximum hop count is used as the trigger condition.

The experimental results shown in Figure 6 indicate that if the maximum hop count is used as the trigger threshold, the late-stage CH disconnection rate may still increase substantially; furthermore, no stable correspondence exists between the disconnection surge and the maximum hop count. As a result, global reclustering cannot be activated reliably at the critical time. By contrast, when the tail ratio of the hop-count distribution is used as the trigger metric, global reclustering can be activated more robustly and can effectively suppress late-stage connectivity degradation.

Based on the above analysis, the sharp rise in late-stage CH disconnection rate should be interpreted as a structural problem observed in the representative cases examined in this study, rather than as an isolated anomaly caused by a single random link failure. More intense flow fields push the system toward severe connectivity degradation earlier; even under relatively mild flow fields, however, long-term drifting can still gradually drive the backbone into a fragile and mismatched state. This observation explains why an additional global correction mechanism is still needed even when local HELP-based repair remains effective during the early and middle stages of operation.

To make global reclustering both predictive and calibratable, this study replaces average and maximum hop count with the hop-distribution tail ratio $\rho_{tail}\left(t\right)$ as the trigger criterion and combines it with a persistence condition to avoid false triggering. The experimental results show that this design contributes to both overall CH-connectivity improvement and late-stage degradation suppression. Without conditional global reclustering, local HELP-based repair can still address short-term disconnection events, but accumulated drift gradually degrades the backbone and increases the CH-disconnection level. By introducing $\rho_{tail}\left(t\right)\;$ based conditional global reclustering, the system preserves event-driven local repair as the primary maintenance architecture while supplementing it with a necessary global topology-correction capability under prolonged drifting.

In this context, $C_{reach}\left(t\right)$ should be understood as the set of Cluster Heads (CHs) that maintain a valid multi-hop uplink path to the Base Station (BS), rather than being restricted to CHs with direct single-hop connectivity. Consequently, the hop count $h_{c}\left(t\right)$ for nodes within this set can be any valid value greater than or equal to 1.

#### 5.3.4. Quantitative Late-Stage Evaluation of Conditional Global Reclustering

Although local repair can respond to short-term disconnection events, the full-period mean CH-disconnection rate remains 9.13% in Proposed-Local when conditional global reclustering is disabled. After enabling conditional global reclustering, Proposed-Hybrid reduces the full-period mean CH-disconnection rate to 5.15%. This indicates that the global corrective layer contributes not only to late-stage peak suppression but also to the reduction of overall CH-level disconnection under prolonged drifting.

To evaluate this effect more directly, this study reports late-stage metrics over the final third of the simulation period. Because each simulation run lasts 60 h, the final third corresponds to hours 41–60. For each independent run, the full-period mean CH-disconnection rate, the final-third mean CH-disconnection rate, and the late-stage 95th-percentile CH-disconnection peak are computed. Confidence intervals are computed across the 10 independent runs, and the key differences between Proposed-Local and Proposed-Hybrid are evaluated using Welch’s two-sample *t*-tests.

All values are CH-disconnection rates reported as mean ± 95% confidence interval. For each method, the confidence interval is computed across 10 run-level summary values. The confidence intervals in Table 7 are calculated across independent simulation runs. For each run, the corresponding metric is first summarized as a run-level value, and the reported mean and 95% confidence interval are then computed across the 10 run-level values.

The results in Table 8 show that conditional global reclustering improves both full-period and late-stage CH-connectivity behavior relative to Proposed-Local. Compared with Proposed-Local, Proposed-Hybrid reduces the full-period mean CH-disconnection rate from 9.13% to 5.15%. In the final third of the simulation, Proposed-Hybrid further reduces the mean CH-disconnection rate from 16.32% to 8.51%. It also reduces the late-stage 95th-percentile peak from 34.43% to 17.21%. The Welch’s two-sample *t*-test results in Table 8 further show that the reductions in the final-third mean CH-disconnection rate and the late-stage 95th-percentile peak are statistically significant, with *p*-values of $2.89\times 10^{-6}$ and $1.36\times 10^{-5}$, respectively.

Although DARCR shows slightly lower final-third mean CH-disconnection rate and late-stage 95th-percentile peak than Proposed-Hybrid, this behavior is consistent with its fixed-period global reorganization strategy. Because DARCR periodically refreshes the network topology, accumulated late-stage backbone degradation can be regularly corrected. In contrast, Proposed-Hybrid does not rely on routine global refresh; it prioritizes event-driven local repair and activates global reclustering only when persistent topology-quality degradation is detected. Therefore, the slightly lower late-stage metrics of DARCR reflect the benefit of scheduled global topology refresh, whereas Proposed-Hybrid achieves a lower full-period mean CH-disconnection rate while preserving a local-repair-first maintenance architecture.

### 5.4. Control Overhead and Control-Plane Cost Index of Proposed-Hybrid

In the control-plane cost accounting, LowTx represents intra-cluster control transmissions, including CH declarations, member join messages, handover announcements, and Node-HELP request/reply messages within $R_{c1\;}$. HighTx represents backbone-level control transmissions, including CH-HELP requests, candidate upstream replies, route-validation messages, upstream-confirmation exchanges, route-recovery notifications, loop-check/path-validation messages, and topology-quality summaries used for $\rho_{tail}\left(t\right)$ evaluation when transmitted as explicit control messages. When topology-quality summaries are piggybacked on normal CH reporting, they are covered by the corresponding reporting or route-validity transmission category rather than modeled as a separate flooding process.

Compared with periodic reorganization, event-driven maintenance may involve multiple rounds of local broadcasting, querying, and repair procedures within a round. In the hybrid design, additional control overhead may also be incurred when the conditional global reclustering mechanism is triggered. Therefore, the number of control packets and their associated transmission burden must be evaluated independently. In this study, LowTx denotes intra-cluster control transmission (approximately within $R_{c1}$), whereas HighTx denotes inter-cluster or backbone control transmission (approximately within $R_{c2}$).

To compare the control burden of different maintenance mechanisms, this study further defines a control-plane cost index by weighting LowTx and HighTx:

(17) $$C_{cp}(w_{H})=LowTx+w_{H}\cdot HighTx,$$

It should be emphasized that $C_{cp}$ is not the residual-energy model used for node-energy updates. The residual-energy state defined in Section 3.2 is updated using normalized operation costs, where a long-range transmission has a simplified 3:1 cost ratio relative to an intra-cluster transmission. By contrast, $C_{cp}$ is a reporting-level control-plane burden index used only to compare the relative overhead of maintenance mechanisms.

The coefficient 256 is therefore not interpreted as the actual per-packet energy-deduction ratio in the simulator. Instead, it is adopted as a penalty weight to emphasize that long-range backbone control transmissions are more expensive and more disruptive than intra-cluster control transmissions. This penalty weight is inspired by the fourth-power distance-scaling concept of the first-order radio-energy model. Since $R_{c2}/R_{c1}=20/5=4$, the corresponding distance-scaling factor is $\left(R_{c2}/R_{c1}{)}^{4}=256\right.$. Thus, $C_{cp}$ should be interpreted only as a comparative control-plane transmission-burden metric, not as total physical energy consumption or as the summed residual-energy depletion of all nodes.

**Figure 7:**
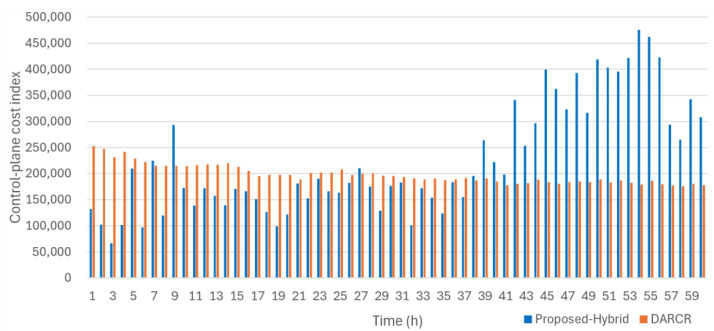
Comparison of control-plane cost index between Proposed-Hybrid and DARCR.

Figure 7 compares the control-plane cost index of Proposed-Hybrid and DARCR. Because DARCR adopts periodic reorganization, its control-plane curve is relatively smooth and shows less round-to-round variation. Proposed-Hybrid, by contrast, incurs higher control cost during periods of topology degradation, concentrated repair events, and occasional conditional global reclustering. According to the experimental statistics, the mean control-plane cost index $C_{cp}(256)$ of Proposed-Hybrid is 13,531,630, whereas that of DARCR is 11,882,826, indicating that the former is approximately 13.9% higher than the latter.

Together with the statistical analysis in Section 5.3.4, the node-number scalability analysis in Section 5.6, and the parameter-sensitivity analysis in Section 5.7, this control-plane cost index provides a practical assessment of the maintenance burden of the proposed framework under the adopted simulation setting.

### 5.5. Supplementary Comparison with a Literature-Grounded MBC-Based Baseline and HEED-Based Baselines

To broaden the comparative context beyond DARCR, this section presents supplementary comparisons with two literature-grounded baselines under the same simulation setting: an adapted mobility-based clustering baseline, denoted as MBC-Based, and an adapted HEED-Based clustering baseline. The supplementary MBC-Based baseline was implemented primarily with reference to the mobility-based clustering protocol proposed by Deng et al. [26], which represents a mobility-aware clustering approach for wireless sensor networks with mobile nodes. In this implementation, the core mobility-based clustering concepts were preserved, including energy- and mobility-aware cluster-head election, estimated-connection-time (ECT)-based member association, and ECT-aware backbone parent selection.

Specifically, CH candidates are ranked according to normalized residual energy, mobility stability, and estimated BS reachability. Member nodes select CHs based on ECT, CH residual energy, and distance, while CH backbone routing selects parent CHs according to ECT, parent residual energy, and hop-level preference. These simulation-level details were further adapted to the continuous-drift sea-surface reporting scenario and the unified evaluation environment considered in this study.

In addition to MBC-Based, an adapted HEED-Based baseline is included as an energy- and communication-cost-aware clustering comparator. HEED, proposed by Younis and Fahmy [17], is a classical distributed clustering protocol in which CH selection is primarily guided by residual energy, with intra-cluster communication cost used as a secondary clustering parameter. In the adapted baseline used in this study, the core HEED principle is retained: CH election follows residual-energy and communication-cost awareness, while member nodes associate with reachable CHs according to local communication cost. To make the baseline compatible with the sea-surface reporting scenario considered in this work, CH-to-BS and CH-to-CH backbone construction are implemented under the same $R_{c1}$ and $R_{c2}$ based topology-level communication framework used in the simulations.

**Figure 8:**
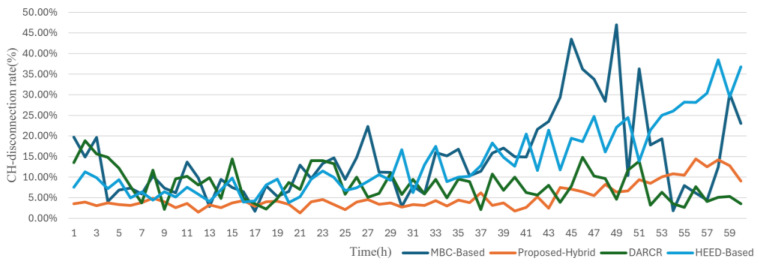
Comparison of CH-disconnection rates among MBC-Based, HEED-Based, Proposed-Hybrid, and DARCR.

**Figure 9:**
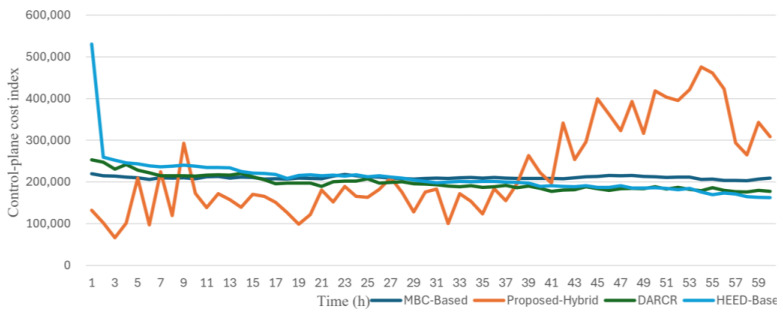
Control-plane cost-index comparison among MBC-Based, HEED-Based, Proposed-Hybrid, and DARCR.

To ensure a consistent comparison, DARCR, MBC-Based, HEED-Based, and Proposed-Hybrid are evaluated under the same sea-surface drifting simulation environment, including the same node deployment, communication radii, base-station placement, ocean-current forcing, simulation duration, random-seed policy, and evaluation metrics. The proposed method is compared with MBC-Based, HEED-Based, and DARCR in terms of CH-disconnection rate, as shown in Figure 8, and control-plane cost index, as shown in Figure 9.

Under the continuous-drift sea-surface reporting scenario considered in this study, the literature-grounded MBC-Based baseline yields a full-period mean CH-disconnection rate of 14.57 ± 0.45%, a final-third mean CH-disconnection rate of 22.39 ± 2.47%, and a late-stage 95th-percentile peak of 50.48 ± 2.32%. In comparison, Proposed-Hybrid achieves 5.15 ± 0.42%, 8.51 ± 1.49%, and 17.21 ± 2.61% for the same three metrics. These results indicate that, under the adopted cluster/backbone reporting model and simulation settings, the proposed maintenance framework provides more effective CH-level connectivity preservation than the adapted MBC-Based baseline.

The hop-count result further clarifies the behavior of the MBC-Based baseline. MBC-Based achieves a mean average hop of 3.2561, which is close to those of DARCR, HEED-Based, and Proposed-Hybrid under the same reference setting. Therefore, the weaker CH-level connectivity preservation of MBC-Based is not primarily attributable to excessive route depth, but rather to insufficient topology-maintenance capability under continuous drift.

In terms of control-plane burden, the MBC-Based baseline has a lower control-plane cost index of 12,582,347. This result indicates that the proposed method incurs additional control-plane overhead in exchange for improved connectivity preservation and sub-step-level repair responsiveness. Accordingly, the primary advantage of the proposed method lies in maintaining CH-level connectivity under continuous drift, whereas the adapted MBC-Based baseline incurs lower control-plane overhead under the present evaluation setting.

The adapted HEED-Based baseline further provides a clustering-oriented comparison based on residual-energy and communication-cost-aware CH selection. For the same three metrics, HEED-Based yields a full-period mean CH-disconnection rate of 13.74 ± 1.21%, a final-third mean CH-disconnection rate of 23.42 ± 2.54%, and a late-stage 95th-percentile peak of 41.48 ± 4.88%. These values are higher than those of Proposed-Hybrid, which achieves 5.15 ± 0.42%, 8.51 ± 1.49%, and 17.21 ± 2.61% for the same three metrics. This result suggests that residual-energy and communication-cost-aware clustering alone is insufficient to provide robust CH-level topology maintenance under continuous sea-surface drift.

The hop-count result also supports this interpretation. HEED-Based achieves a mean average hop of 3.2385, which is close to those of MBC-Based, DARCR, and Proposed-Hybrid under the same reference setting. Similar to MBC-Based, the main limitation of HEED-Based is therefore not excessive route depth, but the difficulty of maintaining CH-level backbone connectivity as the topology continuously evolves under sea-surface drift.

In terms of control-plane burden, HEED-Based obtains a control-plane cost index of 12,632,385 under the adopted accounting setting. This value is comparable to the control-plane cost index of Proposed-Hybrid rather than being substantially lower. However, HEED-Based still exhibits weaker CH-level connectivity preservation and a higher late-stage peak. This suggests that the advantage of Proposed-Hybrid is not solely attributable to a larger number of control transmissions, but to the use of event-driven repair and topology-maintenance procedures that more directly address drift-induced disconnection and late-stage topology degradation.

Taken together, the supplementary MBC-Based and HEED-Based baselines show that clustering-oriented baseline designs are insufficient to achieve the same self-healing topology-maintenance performance as Proposed-Hybrid under the adopted continuous-drift sea-surface reporting scenario. MBC-Based incorporates mobility-aware clustering and ECT-aware association, while HEED-Based incorporates residual-energy and communication-cost-aware clustering. However, clustering-oriented maintenance alone does not provide the same level of drift-induced topology recovery as the proposed event-driven maintenance framework. Consequently, their CH-disconnection rates and late-stage peaks remain higher than those of Proposed-Hybrid. This comparison should be interpreted as a supplementary evaluation under the adopted simulation setting.

### 5.6. Scalability Analysis with Different Node Numbers

**Figure 10:**
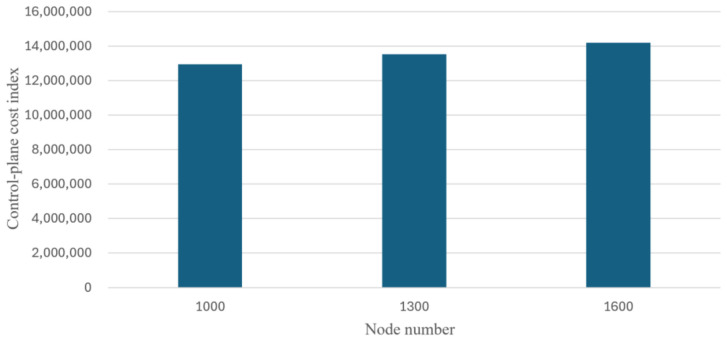
Control-plane cost index under different node-number settings.

To examine how deployment size affects maintenance burden and connectivity, we compare three representative settings: 1000, 1300, and 1600 nodes under the same drifting environment and simulation duration. The 1300-node setting corresponds to the reference configuration used in Section 5.1, Section 5.2, Section 5.3, Section 5.4 and Section 5.5, whereas the 1000-node and 1600-node settings are introduced only for scalability analysis under lower and higher node-density conditions. Therefore, the 1300-node results in this subsection are identical to the main results reported earlier. Figure 10 shows the control-plane cost-index behavior under different node-number settings. The aggregate control-plane cost index increases from 12,946,094.11 for 1000 nodes to 13,531,630.45 for 1300 nodes and further to 14,182,695.50 for 1600 nodes, corresponding to increases of approximately 4.5% and 4.8%, respectively. This indicates that the overall control-plane maintenance burden becomes larger as the deployment size increases. However, when normalized by the number of nodes, the aggregate control-plane cost index per node decreases from 12,946.09 to 10,408.95 and further to 8864.18. This suggests that although denser deployments incur a higher aggregate control-plane cost index, the control-plane transmission burden is distributed across more nodes, resulting in a lower per-node average control-plane burden.

**Figure 11:**
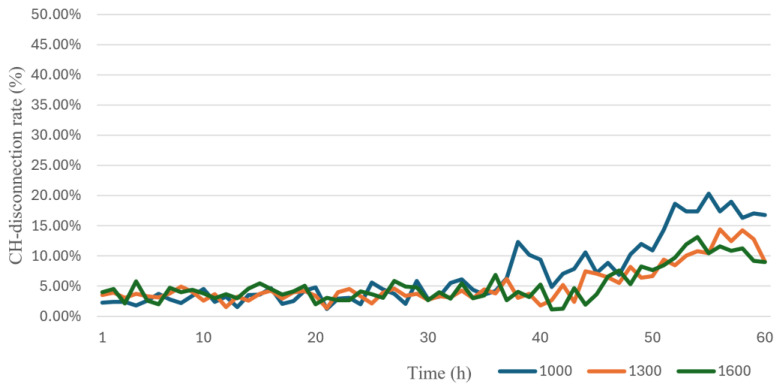
Comparison of CH disconnection rates under different node-number settings.

Figure 11 compares the CH disconnection-rate behavior under the same three settings. The average CH disconnection rates are 7.01%, 5.15%, and 5.14% for 1000, 1300, and 1600 nodes, respectively. These results suggest that denser deployment improves connectivity robustness under drifting conditions, since a larger number of nodes provides more opportunities for maintaining or restoring effective reporting paths. However, the improvement from 1300 to 1600 nodes is smaller than that from 1000 to 1300 nodes, indicating that the connectivity gain does not increase proportionally with node number. Within the tested node-number range, the improvement from 1300 to 1600 nodes is limited, suggesting a possible saturation trend rather than a proportional gain with further node increases.

### 5.7. Parameter Sensitivity Analysis

To examine whether the proposed method depends excessively on a specific parameter configuration, this section evaluates the sensitivity of key parameters related to stability triggering and backbone reachability. The analysis focuses on two aspects: network stability, measured by CH-disconnection rate and hop-count behavior; and control-plane overhead, measured by the control-plane cost index and maintenance-related control activity.

#### 5.7.1. Stability-Threshold Sensitivity

Since $S_{THRESHOLD}$ directly controls the triggering timing of stability-based handover, an excessively low threshold may delay necessary preventive repair, whereas an excessively high threshold may lead to overly aggressive maintenance behavior and increased control-plane burden. To evaluate this trade-off, three representative values, $S_{THRESHOLD}=0.1$, 0.2, and 0.3, are compared under the same 1300-node reference configuration. The preliminary simulations show that the corresponding average CH-disconnection rates are 5.87%, 5.15%, and 6.32%, respectively. Among these settings, $S_{THRESHOLD}=0.2$ yields the lowest average CH-disconnection rate, indicating a better balance between preventive responsiveness and maintenance stability under the adopted simulation conditions. Therefore, this value is used as the reference setting in the main experiments, but it should not be interpreted as a universally optimal threshold for all deployment environments.

#### 5.7.2. Backbone Communication-Radius Sensitivity

Because $R_{c2}=20$ km is an idealized backbone connectivity radius inherited from the DARCR simulation setting rather than a guaranteed physical communication range under all marine VHF conditions, an additional sensitivity analysis is conducted by reducing $R_{c2}$ to 15 km and 10 km.

The backbone communication radius $R_{c2}$ affects the availability of feasible upstream CHs and therefore influences route stability, recovery difficulty, hop depth, and control-plane burden. To examine the sensitivity of the proposed mechanism to this topology-level communication assumption, $R_{c2}=10$, 15, and 20 km are compared while the intra-cluster communication radius $R_{c1}=5\;km$ and the other simulation settings are kept unchanged. The 20 km setting corresponds to the reference configuration used in the main experiments, whereas the 10 km and 15 km settings represent more conservative backbone-connectivity assumptions.

Table 9 summarizes the $R_{c2}$ sensitivity results for Proposed-Hybrid and DARCR. The results show that increasing $R_{c2\;}$ generally improves backbone reachability and reduces route depth. For DARCR, the mean CH-disconnection rate decreases from 22.04% at $R_{c2}=10\;km$ to 12.56% at $R_{c2}=15\;km$ and 8.15% at $R_{c2}=20\;km$. The mean average hop also decreases from 7.092 to 4.824 and 3.233, respectively. A similar connectivity trend is observed for Proposed-Hybrid, whose mean CH-disconnection rate decreases from 24.42% at $R_{c2}=10\;km$ to 9.10% at $R_{c2}=15\;km$ and 5.15% at $R_{c2}=20\;km$. Its mean average hop decreases from 7.103 to 4.375 and 3.186, respectively. These results confirm that the assumed backbone communication radius has a strong effect on CH-level reachability and route depth.

Mean maximum hop denotes the average maximum-hop value across independent simulation runs.

At $R_{c2}=10$ km, Proposed-Hybrid does not outperform DARCR in mean CH-disconnection rate. This indicates that when the backbone communication radius is too restrictive, many CHs cannot find feasible upstream candidates, and local repair alone cannot fully compensate for insufficient backbone reachability. Under this setting, the network tends to form deeper and more fragile multi-hop routes, as reflected by the mean average hop of 7.103 and the mean maximum hop of 19.50 for Proposed-Hybrid. Therefore, the benefit of event-driven local repair is limited when the underlying topology is severely constrained by a short $R_{c2}$.

When $R_{c2}$ increases to 15 km and 20 km, Proposed-Hybrid achieves lower CH-disconnection rates than DARCR. At $R_{c2}=15$ km, Proposed-Hybrid reduces the mean CH-disconnection rate from 12.56% in DARCR to 9.10%. At $R_{c2}=20$ km, it further reduces the rate from 8.15% in DARCR to 5.15%. These results suggest that once sufficient backbone reachability is available, the proposed CH-HELP, Node-HELP, route inheritance, loop-free upstream reselection, and conditional global reclustering mechanisms can more effectively preserve CH-level connectivity than the periodic maintenance strategy of DARCR.

The $C_{cp}$ trend should be interpreted together with the accumulated HighTx burden rather than as a direct physical energy-consumption metric. For Proposed-Hybrid, $C_{cp}$ exhibits a non-monotonic pattern: 9,063,175 at $R_{c2}=10$ km, 8,538,110 at $R_{c2}=15$ km, and 13,531,630 at $R_{c2}=20$ km. This indicates that $R_{c2}=15$ km yields the lowest control-plane burden under the tested settings. At $R_{c2}=10$ km, the backbone is too restrictive, producing frequent failed recovery attempts and long fragile routes. At $R_{c2}=20$ km, connectivity and hop depth improve, but the larger backbone range allows more successful backbone-level interactions, including route setup, upstream confirmation, route-validity propagation, repair-related route updates, and conditional correction. Since $C_{cp\;}$ gives a high penalty to HighTx, these additional backbone-level control exchanges can increase the overall cost index even when the CH-disconnection rate decreases.

The DARCR results provide a useful contrast. Unlike Proposed-Hybrid, DARCR does not include the proposed CH-HELP and Node-HELP mechanisms. Nevertheless, its $C_{cp\;}$ increases monotonically from 4,258,515 at $R_{c2}=10$ km to 7,457,954 at $R_{c2}=15$ km and 11,882,826 at $R_{c2}=20$ km. At the same time, its mean average hop decreases from 7.092 to 4.824 and 3.233. This trend supports the interpretation that increasing $R_{c2\;}$ improves backbone reachability and reduces route depth, but also increases the amount of backbone-level route construction, topology refresh, and recovery-related control activity. Therefore, the non-monotonic $C_{cp}$ pattern of Proposed-Hybrid should be understood as the combined effect of two factors: failed-recovery pressure when $R_{c2}$ is too small and HighTx-dominated backbone maintenance burden when $R_{c2}$ is large.

Overall, the $R_{c2}$ sensitivity analysis shows that the proposed method is sensitive to the assumed topology-level backbone communication radius. A larger $R_{c2\;}$ generally improves CH-level connectivity and reduces hop depth, but it does not necessarily reduce the control-plane cost index. The 20 km setting used in the main experiments should therefore be interpreted as an idealized backbone-connectivity assumption for evaluating routing-maintenance behavior, rather than as a universal physical communication range for all sea-surface deployments.

#### 5.7.3. Robustness of the Control-Plane Cost Comparison Under Different HighTx Penalty Weights

To evaluate whether the control-plane comparison depends excessively on the selected HighTx penalty weight, Table 10 reports $C_{cp}(w_{H})$ under three representative weighting settings, $w_{H}$ = 3, 10, and 256, together with the raw LowTx and HighTx counts.

The sensitivity results show that the interpretation of control-plane overhead depends on the selected HighTx penalty weight. When $w_{H}=3$ or $w_{H}=10$, LowTx still contributes substantially to the cost index, and MBC-Based yields the lowest $C_{cp}\left(w_{H}\right)$ because it generates fewer LowTx control transmissions. When $w_{H}=256$, HighTx becomes the dominant cost component, and the differences among methods are mainly determined by backbone-level control transmissions. Under this strict HighTx-penalty setting, Proposed-Hybrid produces the highest $C_{cp}(256)$, indicating that its improved CH-connectivity performance is achieved at the cost of higher backbone-level maintenance overhead. Therefore, the proposed method should be interpreted as a connectivity-oriented self-healing mechanism rather than a minimum-overhead routing scheme.

## 6. Conclusions, Limitations, and Future Work

This study proposes an event-/condition-triggered, local-repair-first maintenance mechanism for sea-surface drifting wireless sensor networks. By integrating dual-threshold CH handover, CH-HELP, Node-HELP, and conditional global reclustering, the framework aims to reduce maintenance gaps while constraining long-term backbone degradation under continuous drift.

Through simulation experiments driven by realistic current-dominant flow–wind data from the Taiwan Strait, this study quantitatively compares the proposed mechanism with DARCR and reaches the following conclusions.

Improved connectivity and reliability. The full Proposed-Hybrid method achieves a mean CH-disconnection rate of 5.15%, lower than the 8.15% observed for DARCR and the 14.57% observed for the adapted MBC-Based baseline. In contrast, the local-only Proposed-Local configuration without conditional global reclustering yields a higher CH-disconnection rate of 9.13%, showing that local repair alone is insufficient to maintain long-term CH-level connectivity under prolonged drift. Compared with Proposed-Local, Proposed-Hybrid further reduces the final-third mean CH-disconnection rate from 16.32% to 8.51% and the late-stage 95th-percentile peak from 34.43% to 17.21%. These results indicate that conditional global reclustering functions as an effective corrective layer for suppressing accumulated backbone degradation.

The proposed hybrid strategy does not claim universal dominance over DARCR in every late-stage indicator. Instead, it provides a lower full-period CH-disconnection rate and mitigates the severe late-stage degradation observed in the local-only version, while DARCR remains competitive in some late-stage metrics because of its periodic global reorganization.

Although DARCR, the MBC-Based baseline, and the adapted HEED-Based baseline provide controlled comparisons under identical simulation conditions, these baselines were implemented within the same simulation framework. Future work should include independent implementations from additional routing families, such as opportunistic, geographic, or DTN-style marine routing, to further assess the generality of the proposed maintenance framework.

This study adopts a binary disk connectivity model for topology-level evaluation. Although this abstraction enables controlled comparison of routing-maintenance mechanisms, it does not explicitly model probabilistic marine VHF link quality, antenna-height limitations, sea-surface two-ray propagation, wave shadowing, sea-state-dependent fading, receiver sensitivity, or interference. Therefore, the reported connectivity performance should be interpreted as topology-level behavior under the assumed communication radii rather than as a direct prediction of physical VHF link availability. Future work will extend the simulator by incorporating probabilistic or propagation-loss-based marine link models to evaluate the proposed maintenance mechanism under more realistic physical-layer conditions.

Routing depth remains bounded under the adopted hop-based delay proxy. During 60 h of dynamic drifting, the average hop count remains mostly below 4, while the maximum hop count is kept below 10. This suggests that route elongation is constrained in terms of hop depth, although actual end-to-end latency was not directly modeled at the MAC/PHY level.

Trade-off between control overhead and repair responsiveness. The control-plane cost index of Proposed-Hybrid is approximately 13.9% higher than that of DARCR. This result indicates that the hybrid event-driven maintenance strategy achieves a lower CH disconnection rate and improved sub-step-level repair responsiveness at the cost of higher control-plane transmission overhead.

The $R_{c2}$ sensitivity analysis further shows that the backbone communication-radius assumption strongly affects both connectivity and control-plane burden. Increasing $R_{c2}$ generally reduces CH-disconnection rate and hop depth for both Proposed-Hybrid and DARCR, confirming that backbone reachability is a key factor in continuous-drift sea-surface WSNs. However, the control-plane cost index does not decrease monotonically with $R_{c2}$. For Proposed-Hybrid, the lowest $C_{cp}$ occurs at the intermediate $R_{c2}$ = 15 km setting, whereas $R_{c2}$ = 20 km provides the best connectivity but also produces the highest control-plane cost index. This result indicates that improved backbone reachability can reduce failed recovery pressure, but may also increase HighTx-dominated route setup, route-validity propagation, and backbone-maintenance interactions. Therefore, $R_{c2}$ should be regarded as a topology-level modeling assumption that affects the connectivity-cost trade-off, rather than as a universally optimal communication range.

In addition to communication-radius sensitivity, the handover-successor screening rule also remains a future extension. The present implementation does not impose an additional hard feasibility-aware successor-screening rule, such as requiring $S_{self}(n,t)>S_{self}(C,t)+\Delta S$ together with additional energy, distance, and route-validity constraints. Such a rule would define a new handover variant and is left for future evaluation.

## Figures and Tables

**Table 1:** Mechanism-level comparison between DARCR and the proposed framework.

Aspect	DARCR	Proposed Framework
Maintenance philosophy	Drift-aware clustering with periodic topology refresh	Event-/condition-triggered, local-repair-first maintenance
Primary maintenance trigger	Fixed-time or fixed-round refresh	Disconnection, instability, role change, or topology-quality degradation
Maintenance granularity	Mainly network-wide reorganization	Local repair first; global reclustering only under sustained degradation
Response to intra-round failures	Mainly aligned with the next scheduled refresh	Repair can be initiated within the same observation sub-step
Cluster-head replacement	Coupled with periodic reorganization	Dual-threshold CH handover using both energy and stability conditions
Backbone repair	Self-recovery routing after disconnection	CH-HELP with upstream reselection and route validation
Member-node recovery	Embedded in the routing/clustering process	Node-HELP for isolated member-node reattachment
Route continuity after CH change	Backbone refresh or reconstruction	Route inheritance with local upstream validation
Loop prevention during repair	Not emphasized as a dedicated mechanism	Pre-selection exclusion and run-time loop detection
Long-term topology-quality control	Mainly periodic refresh	$\mathrm{Uses}\;\rho_{tail}(t)$ based conditional global reclustering
Global reclustering role	Routine maintenance mechanism	Low-frequency safety valve
Control-plane temporal pattern	More regular because of scheduled maintenance	Less dependent on fixed maintenance timing
Expected trade-off	Lower control volatility but slower local response	Faster recovery and lower CH disconnection, with higher control overhead

**Table 2:** Summary of main notation and parameters.

Category	Symbol/Notation	Description	Symbol/Notation	Description
Network model	A	Target monitoring area	L, W	Length and width of the monitored area
	N	Total number of sensor nodes	$n_{i}$	Sensor node *i*
	B	Set of base stations	BS	Base station
	$P_{i\left(t\right)}$	Position of node *i* at time *t*		
Mobility model	$u_{i\left(t\right)},\;v_{i\left(t\right)}$	Horizontal velocity components of node *i*	$V_{i\left(t\right)}$	Speed magnitude of node *i*
	∆t	Simulation time step		
Communication model	$R_{s}$	Sensing radius		
	$R_{c1}$	Intra-cluster communication radius	$R_{c2}$	Backbone communication radius
Energy model	E(i)	Residual energy of node *i*	$E_{0}$	Initial normalized energy
	$E_{RX}$	Receive cost per operation	$E_{TX-low}$	Intra-cluster transmission cost per operation
	$E_{TX-high}$	Long-range transmission cost per operation		
Handover trigger	$E_{TRIGGER}$	Residual-energy warning threshold	∆E	
Stability and routing	$S_{self}$	Self-stability of a node	$S_{link}$	Link stability between two nodes
	$S_{THRESHOLD}$	Self-stability threshold	$S_{up}$	Upstream-validity state
	H(X)	Hop count from node *X* to a base station	$H_{old}$	Original hop count before route update
	$∆H_{max}$	Maximum allowed hop-count increase when a finite previous valid hop count is available	Score(C→U)	Upstream-selection score from CH *C* to candidate *U*
	$w_{1},\;w_{2}$	Weights for link stability and hop penalty	$V_{ref}$	Reference speed scale used in the self-stability calculation.
	$V_{0}$	Speed-scale constant used in the link-stability calculation.		
Global reclustering	$C_{reach(t)}$	Set of reachable CHs whose summaries reach the BS	$h_{\left(c,t\right)}$	Hop count of CH *c* at time *t*
	$H_{tail}$	Long-hop threshold	D(t)	Topology-degradation observation indicator at time *t*.
	$\theta_{tail}$	Tail-ratio threshold for conditional global reclustering	$T_{recluster\_min}$	Minimum interval between two global reclustering operations
	$\rho_{tail}(t)$	Ratio of reachable CHs whose hop count exceeds $H_{tail}$		
Control cost	LowTx	Intra-cluster control transmission count	HighTx	Backbone-level control transmission count
	$C_{cp}$	Control-plane cost index		

**Table 3:** Computational and control-message complexity of the proposed maintenance components.

Component	Computation Complexity	Control-Message Complexity	Scope
CH handover evaluation	$O(m_{C})$	$O(m_{C})$	Current cluster members
Route inheritance validation	$O(D_{max})$	$O(D_{max})$	Upstream chain
CH-HELP upstream reselection	$O(d_{CH}(C)D_{max})$	$O(d_{CH}(C)D_{max})$	Neighboring CHs
Node-HELP reattachment	$O(d_{node}(i))$	$O(d_{node}(i))$	Nearby CHs
Conditional global reclustering	$O(NK+K^{2})$	O(N+K)	Network-wide, low frequency

**Table 4:** Simulation parameters.

Category	Parameter	Symbol	Value
Environment	Area	A	(100 × 100) km
	Total nodes	N	1300
	Simulation duration	-	60 h
	Round duration	-	1 h
	Sub-step duration	Δt	10 min
	Sub-steps per round	-	6
	Flow field source	-	Copernicus Marine Service, (GLOBAL_ANALYSISFORECAST_PHY_001_024)
	Wind-field source	-	Copernicus Marine Service, Global Ocean Hourly Sea Surface Wind and Stress from Scatterometer and Model
	Ocean-current spatial resolution	-	1/12° × 1/12°
	Wind-field spatial resolution	-	0.125° × 0.125°
	Temporal resolution	-	Hourly
	Spatial matching method	-	Nearest-neighbor grid-point matching followed by local statistical correction
	Temporal matching method	-	Hourly timestamp matching
Communication	Sensing radius	$R_{s}$	2 km
	Intra-cluster communication radius	$R_{c1}$	5 km
	Backbone communication radius	$R_{c2}$	20 km
Weighting	Drift-combination weight	(f)	0.97 (current-dominant weighting factor)
	Backoff weights	*T*_1_–*T*_5_	0.0833, 0.2500, 0.3333, 0.1667, 0.1667
	Link-score weights	$w_{1}$ $,w_{2}$	1.0, 0.3
Normalized energy model	Initial normalized energy	$E_{0}$	5
	Receive cost per operation	$E_{RX}$	0.0005
	Intra-cluster Tx cost per operation	$E_{TX-low}$	0.0010
	Long-range Tx cost per operation	$E_{TX-high}$	0.0030
Maintenance	Energy trigger threshold	$E_{TRIGGER}$	2.5 normalized energy units
	Energy differential threshold	∆E	1.5 normalized energy units
	Stability threshold	$S_{THRESHOLD}$	0.2
	Maximum hop increase	$∆H_{max}$	3
	Long-hop threshold	$H_{tail}$	6
	Tail-ratio threshold	$\theta_{tail}$	0.15
	Minimum global reclustering interval	$T_{recluster\_min}$	20 rounds
	Reference speed scale for self-stability	$V_{ref}$	1.0 m/s
	Speed-scale constant for link stability	$V_{0}$	0.5 m/s

**Table 5:** Direct CH-disconnection comparison between DARCR and Proposed-Hybrid.

Method	Full-Period Mean CH-Disconnection Rate	Final-Third Mean CH-Disconnection Rate	Late-Stage 95th-Percentile Peak
DARCR	8.15±0.44%	6.89±0.64%	16.40±0.83%
Proposed-Hybrid	5.15±0.42%	8.51±1.49%	17.21±2.61%
Welch’s two-sample *t*-test *p*-value	$1.43\times 10^{-9}$	0.0437	0.5178

**Table 6:** Direct route-depth and control-cost comparison between DARCR and Proposed-Hybrid.

Method	Mean Average Hop	Mean Maximum Hop	$C_{cp}$ (256)
DARCR	3.2398 ± 0.0247	10.60 ± 0.37	11,882,826
Proposed-Hybrid	3.2415 ± 0.0563	9.60 ± 0.50	13,531,630
Welch’s two-sample *t*-test *p*-value	0.9496	0.00847	8.31 × 10^−6^

**Table 7:** CH-disconnection metrics with confidence intervals.

Method	Full-Period Mean CH Disconnection Rate	Final-Third Mean CH Disconnection Rate	Late-Stage 95th-Percentile Peak
DARCR	8.15 ± 0.44%	6.89 ± 0.64%	16.40 ± 0.83%
Proposed-Local	9.13 ± 0.95%	16.32 ± 2.06%	34.43 ± 5.21%
Proposed-Hybrid	5.15 ± 0.42%	8.51 ± 1.49%	17.21 ± 2.61%

**Table 8:** Statistical comparison between Proposed-Local and Proposed-Hybrid.

Comparison	Metric	Test	*p*-Value
Proposed-Hybrid vs. Proposed-Local	Final-third mean CH-disconnection rate	Welch’s two-sample *t*-test	$2.89\times 10^{-6}$
Proposed-Hybrid vs. Proposed-Local	Late-stage 95th-percentile peak	Welch’s two-sample *t*-test	$1.36\times 10^{-5}$

**Table 9:** $R_{c2}$ sensitivity results of Proposed-Hybrid and DARCR.

Method	$R_{c2}$	Mean CH-Disconnection Rate	Mean $C_{cp}$	Mean Maximum Hop	Mean Average Hop
Proposed-Hybrid	10 km	24.42%	9,063,175	19.50	7.103
	15 km	9.10%	8,538,110	17.00	4.375
	20 km	5.15%	13,531,630	9.428	3.186
DARCR	10 km	22.04%	4,258,515	20.50	7.092
	15 km	12.56%	7,457,954	18.33	4.824
	20 km	8.15%	11,882,826	9.60	3.233

**Table 10:** Sensitivity of the control-plane cost index to HighTx penalty weight.

Method	LowTx	HighTx	$C_{cp}(3)$	$C_{cp}(10)$	$C_{cp}(256)$
DARCR	144,253	45,854	281,815	602,791	11,882,826
MBC-Based	66,635	48,890	213,304	555,530	12,582,347
HEED-Based	228,520	48,453	373,878	713,046	12,632,385
Proposed-Local	301,906	49,406	450,124	795,963	12,949,766
Proposed-Hybrid	158,779	52,238	315,492	681,156	13,531,630

## Data Availability

The ocean-current and wind-field inputs used in this study were processed from Copernicus Marine Service products. The ocean-current input was obtained from GLOBAL_ANALYSISFORECAST_PHY_001_024, and the wind-field input was obtained from the Copernicus Marine sea-surface wind product Global Ocean Hourly Sea Surface Wind and Stress from Scatterometer and Model. The processed environmental inputs used in the simulation were converted into hourly u/v components over the Taiwan Strait region and combined as a current-dominant flow–wind weighted drift field. The detailed reproducibility materials are provided as Supplementary File S1, which is distributed as a compressed package. This package includes a PDF document describing the complete simulation configuration, environmental-data product information, processed input windows, deployment-boundary definitions, base-station positions, parameter settings, control-plane counter definitions, and the random-seed limitation statement. It also includes an Excel workbook containing the run-level numerical result tables and raw-result tables used to support the reported figures and tables. The supplementary MBC-Based and HEED-Based baseline implementation details are provided in Supplementary File S2.

## Supplementary Materials

The following supporting information can be downloaded at: https://www.mdpi.com/article/10.3390/s26123915/s1, Supplementary File S1 contains the reproducibility materials, including a reproducibility document in PDF format and an Excel workbook containing the run-level simulation results used in this study. Supplementary File S2 contains additional implementation details of the baseline protocols and experimental settings.

## Abbreviations

The following abbreviations are used in this manuscript:

WSN	Wireless Sensor Network
CH	Cluster Head
BS	Base Station
DARCR	Drift-Aware Routing and Clustering with Recovery
MBC-Based	Adapted mobility-based clustering baseline
HEED-Based	Adapted HEED-based clustering baseline
HELP	Help-based Local Repair Procedure
ECT	Estimated Connection Time
MAC	Medium Access Control
PHY	Physical Layer

## References

[1] Akyildiz I.F., Su W., Sankarasubramaniam Y., Cayirci E. (2002). Wireless Sensor Networks: A Survey. Comput. Netw..

[2] Xu G., Shen W., Wang X. (2014). Applications of Wireless Sensor Networks in Marine Environment Monitoring: A Survey. Sensors.

[3] Majumder A., Losito M., Paramasivam S., Kumar A., Gatto G. (2024). Buoys for Marine Weather Data Monitoring and LoRaWAN Communication. Ocean Eng..

[4] Lumpkin R., Özgökmen T., Centurioni L. (2017). Advances in the Application of Surface Drifters. Annu. Rev. Mar. Sci..

[5] Frye D., Hamilton A., Grosenbaugh M., Paul W., Chaffey M. (2004). Deepwater Mooring Designs for Ocean Observatory Science. Mar. Technol. Soc. J..

[6] Venkatesan R., Senthilkumar P., Vedachalam N., Murugesh P. (2017). Biofouling and Its Effects in Sensor Mounted Moored Observatory System in Northern Indian Ocean. Int. Biodeterior. Biodegrad..

[7] Barbosa P.N.E.S., White N.M., Harris N.R. (2008). Wireless Sensor Network for Localized Maritime Monitoring. Proceedings of the 22nd International Conference on Advanced Information Networking and Applications—Workshops (AINA Workshops 2008), Ginowan, Japan, 25–28 March 2008.

[8] Wang L., Hong Q.-X. (2025). A Drift-Aware Clustering and Recovery Strategy for Surface-Deployed Wireless Sensor Networks in Ocean Environments. Sensors.

[9] Qing L., Zhu Q., Wang M. (2006). Design of a Distributed Energy-Efficient Clustering Algorithm for Heterogeneous Wireless Sensor Networks. Comput. Commun..

[10] Li C., Ye M., Chen G., Wu J. An Energy-Efficient Unequal Clustering Mechanism for Wireless Sensor Networks. Proceedings of the IEEE International Conference on Mobile Adhoc and Sensor Systems Conference.

[11] Ahmad M., Li T., Khan Z., Khurshid F., Ahmad M. (2018). A Novel Connectivity-Based LEACH-MEEC Routing Protocol for Mobile Wireless Sensor Network. Sensors.

[12] Wood A.D., Stankovic J.A. (2002). Denial of Service in Sensor Networks. Computer.

[13] Akkaya K., Younis M. (2005). A Survey on Routing Protocols for Wireless Sensor Networks. Ad Hoc Netw..

[14] Shahraki A., Taherkordi A., Haugen Ø., Eliassen F. (2020). Clustering Objectives in Wireless Sensor Networks: A Survey and Research Direction Analysis. Comput. Netw..

[15] Heinzelman W.B., Chandrakasan A.P., Balakrishnan H. (2002). An Application-Specific Protocol Architecture for Wireless Microsensor Networks. IEEE Trans. Wirel. Commun..

[16] Daanoune I., Abdennaceur B., Ballouk A. (2021). A Comprehensive Survey on LEACH-Based Clustering Routing Protocols in Wireless Sensor Networks. Ad Hoc Netw..

[17] Younis O., Fahmy S. (2004). HEED: A Hybrid, Energy-Efficient, Distributed Clustering Approach for Ad Hoc Sensor Networks. IEEE Trans. Mob. Comput..

[18] Hussain M.H.A., Mokhtar B., Rizk M.R.M. (2024). A comparative survey on LEACH successors clustering algorithms for energy-efficient longevity WSNs. Egypt. Inform. J..

[19] Del-Valle-Soto C., Rodríguez A., Ascencio-Piña C.R. (2023). A survey of energy-efficient clustering routing protocols for wireless sensor networks based on metaheuristic approaches. Artif. Intell. Rev..

[20] More A., Raisinghani V. (2017). A Survey on Energy Efficient Coverage Protocols in Wireless Sensor Networks. J. King Saud Univ. —Comput. Inf. Sci..

[21] Xu N., Huang A., Hou T., Chen H. (2012). Coverage and Connectivity Guaranteed Topology Control Algorithm for Cluster-Based Wireless Sensor Networks. Wirel. Commun. Mob. Comput..

[22] Wang L., Kao P.-H., Wu M.-T. (2017). Using Partial Coverage Strategy to Prolong Service Time of a Cluster-Based Wireless Sensor Network. J. Internet Technol..

[23] Wang L., Yang J.-Y., Lin Y.-Y., Lin W.-J. (2014). Keeping Desired QoS by a Partial Coverage Algorithm for Cluster-Based Wireless Sensor Networks. J. Netw..

[24] Kim D.-S., Chung Y.-J. Self-Organization Routing Protocol Supporting Mobile Nodes for Wireless Sensor Network. Proceedings of the First International Multi-Symposiums on Computer and Computational Sciences (IMSCCS’06).

[25] Kumar G.S., Vinu P.M.V., Jacob K.P. Mobility Metric Based LEACH-Mobile Protocol. Proceedings of the 2008 16th International Conference on Advanced Computing and Communications.

[26] Deng S., Li J., Shen L. (2011). Mobility-Based Clustering Protocol for Wireless Sensor Networks with Mobile Nodes. IET Wirel. Sens. Syst..

[27] Manjeshwar A., Agrawal D.P. TEEN: A Routing Protocol for Enhanced Efficiency in Wireless Sensor Networks. Proceedings of the 15th International Parallel and Distributed Processing Symposium.

[28] Manjeshwar A., Agrawal D.P. (2002). APTEEN: A Hybrid Protocol for Efficient Routing and Comprehensive Information Retrieval in Wireless Sensor Networks. Proceedings of the 16th International Parallel and Distributed Processing Symposium (IPDPS 2002), Ft. Lauderdale, FL, USA, 15–19 April 2002.

[29] Gupta G., Younis M. Fault-Tolerant Clustering of Wireless Sensor Networks. Proceedings of the 2003 IEEE Wireless Communications and Networking Conference (WCNC 2003).

[30] Choudhary M., Goyal N. (2022). Dynamic Topology Control Algorithm for Node Deployment in Mobile Underwater Wireless Sensor Networks. Concurr. Comput..

[31] Zhao Z., Liu C., Guang X., Zhao Z., Qu W. (2025). A Reliability-Driven Topology Restoration Strategy for Underwater Wireless Sensor Networks in Dynamic Ocean Environments. IEEE Internet Things J..

[32] Özgökmen T.M., Griffa A., Mariano A.J., Piterbarg L.I. (2000). On the Predictability of Lagrangian Trajectories in the Ocean. J. Atmos. Ocean. Technol..

[33] Röhrs J., Christensen K.H., Hole L.R., Broström G., Drivdal M., Sundby S. (2012). Observation-Based Evaluation of Surface Wave Effects on Currents and Trajectory Forecasts. Ocean Dyn..

[34] Zhang J., Teixeira Â.P., Guedes Soares C., Yan X. (2017). Probabilistic Modelling of the Drifting Trajectory of an Object under the Effect of Wind and Current for Maritime Search and Rescue. Ocean Eng..

[35] Jiang J., Han G., Lin C. (2023). A Survey on Opportunistic Routing Protocols in the Internet of Underwater Things. Comput. Netw..

[36] Nkenyereye L., Nkenyereye L., Ndibanje B. (2024). Internet of Underwater Things: A Survey on Simulation Tools and 5G-Based Underwater Networks. Electronics.

