# Supplementary material for: An Event-Driven Self-Healing Routing and Topology Maintenance Mechanism for Surface-Deployed Wireless Sensor Networks in Ocean Environments
Source: Sensors (Basel). 2026 Jun 20;26(12):3915. doi: 10.3390/s26123915 (PMC13307351; doi:10.3390/s26123915)
Supplement: Supplementary file 1 [file sensors-26-03915-s001.zip › Supplementary_File S1/Supplementary_File_S1_Reproducibility_Materials.pdf]

Supplementary File S1
Reproducibility Materials and Simulation Configuration

S1.1. Scope

This file reports the simulation configuration, environmental-data products, processed input windows, deployment geometry, parameter values, and random-seed limitation. Run-level numerical results are provided in the accompanying Excel workbook included in the Supplementary File S1 package.

S1.2. Environmental Data Products

Input	Product name	Product ID	DOI	Resolution used
Ocean current	Global Ocean Physics Analysis and Forecast	GLOBAL_ANALYSISFORECAST_PHY_001_024	10.48670/moi-00016	0.083 deg × 0.083 deg; hourly surface current fields
Wind	Global Ocean Hourly Sea Surface Wind and Stress from Scatterometer and Model	WIND_GLO_PHY_L4_NRT_012_004	10.48670/moi-00305	0.125 deg × 0.125 deg; hourly wind fields

S1.3. Processed Environmental Input Windows

Input	Processed CSV time range	Latitude range	Longitude range
Ocean current	2025-05-01 12:00:00 to 2025-05-04 12:00:00	23.0000 to 26.0000	118.66669 to 120.91669
Wind	2025-05-01 00:00:00 to 2025-05-04 12:00:00	23.0625 to 25.9375	118.6875 to 120.9375

S1.4. Effective Simulation Interval and Temporal Discretization

Item	Value
Start timestamp	2025-05-01 12:00:00
Executed hourly rounds	60 hourly rounds
Hourly environmental timestamps used	2025-05-01 12:00:00 through 2025-05-03 23:00:00
Sub-step length	600 s = 10 min
Sub-steps per hour	6
Position and distance-matrix update frequency	Every 10-minute sub-step
Flow/wind data loading frequency	Once per hourly round, matched by timestamp

S1.5. Deployment Domain and Base-Station Positions

The node-deployment region is a quadrilateral polygon in longitude-latitude space. Its bounding range is Lon. 119.103959 to 120.705829 and Lat. 23.587578 to 25.781000.

Deployment vertex	Longitude	Latitude
P1	120.384000	25.781000
P2	120.705829	25.574306
P3	119.425788	23.587578
P4	119.103959	23.794272

Base station	Internal x coordinate	Internal y coordinate	Derived longitude	Derived latitude
BS1	277.91	158.79	120.279100	24.912100
BS2	235.25	225.02	119.852500	24.249800

S1.6. Simulation Parameters

**Category**	**Parameter**	**Value**	**Description**
Network	N	1300 nodes	Main simulation configuration
Simulation	T_{sim}	60 h	Sixty hourly rounds
Simulation	Δt_{sub}	600 s	Six 10-minute sub-steps per hour
Communication	R_s	2 km	Sensing radius
Communication	R_{c1}	5 km	Member-to-CH and intra-cluster control radius
Communication	R_{c2}	20 km	CH-to-CH and CH-to-BS backbone radius
Drift	f	0.97	u_B = f × flow_u + (1 − f) × wind_u; same for v_B
Drift	distanceThreshold	0.09 × 0.09	Threshold used in compute_U and compute_V routines
Energy	E_0	5	Initial normalized energy
Energy	E_{RX}	0.0005	Receive cost per operation
Energy	$E_{TX\text{-}low}$	0.0010	Short-range transmit cost per operation
Energy	$E_{TX\text{-}high}$	0.0030	Long-range transmit cost per operation
Handover	$E_{TRIGGER}$	2.5	Gate A for energy-triggered CH handover evaluation
Handover	ΔE	1.5	Gate B residual-energy advantage threshold
Handover	$S_{THRESHOLD}$	0.2	Self-stability threshold
Stability	V_{ref}	1.0 m/s	Self-stability reference speed scale
Stability	V_0	0.5 m/s	Link-stability relative-speed scale
Routing	w_1 / W_SLINK	1.0	Link-stability weight in upstream scoring
Routing	w_2 / W_HOP	0.3	Hop-penalty weight in upstream scoring
Routing	ΔH_{max}	3	Maximum relative hop increase when a finite previous valid hop count is available; implemented as MAX_HOP_INCREASE in the source code.
Global reclustering	H_{tail}	6	Long-hop threshold
Global reclustering	θ_{tail}	0.15	Tail-ratio threshold
Global reclustering	$T_{recluster\_min}$	20 rounds	Minimum interval between global reclustering executions
Control accounting	ENABLE_ROUTE_VALID_MONITOR	true	Enables route-valid observation and propagation
Control accounting	COUNT_TX_FOR_ROUTE_VALID_MONITOR	true	Counts local route-update events as HighTx
Control accounting	EXEC_ROUTE_VALID_MONITOR_TX	false	Route-valid monitoring TX is counted but not energy-deducted
Control accounting	EXEC_NEW_CTRL_TX	false	Additional control-plane TX is counted without extra energy deduction
Ablation	DEFAULT_ABLATION_MODE	FULL	Main reported proposed configuration

S1.7. Control-Plane Counters and Reported Metrics

**Metric / counter**	**Definition**
LowTx	Accumulated intra-cluster control transmission count. Data-plane transmissions are not counted in the control-plane TX lists.
HighTx	Accumulated backbone-level control transmission count. Route-monitoring HighTx is counted when COUNT_TX_FOR_ROUTE_VALID_MONITOR = true.
$C_{cp}(w_H)$	LowTx + w_H × HighTx. The run-level Excel workbook reports sensitivity weights w_H = 3, 10, and 256.
CH-disconnection metric	Snapshot-based CH-level route unreachability to a base station, checked through upstream-chain traversal and R_{c2}-based link reachability.
Average hop / maximum hop	Hop-based route-depth indicators over reachable CH routing structures; not MAC/PHY-layer end-to-end delay.
$\rho_{tail}(t)$	Ratio of reachable CHs whose hop count is at least H_{tail}. Unreachable CHs are reflected in CH-disconnection statistics.

S1.8. Random-Seed Record

The simulations used Java runtime pseudorandom initialization for node deployment and random perturbation terms in the backoff process. Manually assigned random seeds were not separately recorded in the original simulation runs. Therefore, an exact random-seed list cannot be provided for the reported runs. Numerical verification is supported through the configuration reported in this file and the accompanying run-level Excel workbook included in the Supplementary File S1 package.
