# Supplementary material for: An Event-Driven Self-Healing Routing and Topology Maintenance Mechanism for Surface-Deployed Wireless Sensor Networks in Ocean Environments
Source: Sensors (Basel). 2026 Jun 20;26(12):3915. doi: 10.3390/s26123915 (PMC13307351; doi:10.3390/s26123915)
Supplement: Supplementary file 1 [file sensors-26-03915-s001.zip › Supplementary_File S2.pdf]

S2.1. Purpose and Scope

This supplementary file provides the detailed implementation rules for the two supplementary clustering-oriented baselines used in the revised manuscript: the adapted mobility-based clustering baseline, denoted as MBC-Based, and the adapted HEED-Based baseline. These details are provided to improve reproducibility and to clarify the equations, parameter values, CH-count control rules, member-association criteria, uncovered-node handling rules, backbone-construction rules, and tie-breaking rules used in the comparative evaluation.

Both baselines were implemented under the same topology-level simulation framework used in the main manuscript. Specifically, the same node deployment, base-station placement, communication radii, environmental drift field, simulation duration, residual-energy accounting model, CH-disconnection metrics, route-depth metrics, and control-plane accounting rules were used for DARCR, MBC-Based, HEED-Based, Proposed-Local, and Proposed-Hybrid. The intra-cluster communication radius is denoted as R_{c1}, and the CH-to-CH / CH-to-BS backbone communication radius is denoted as R_{c2}. In the simulation setting, $R_{c1} = 5$ km and $R_{c2} = 20$ km.

The purpose of these baselines is not to introduce new proposed mechanisms, but to provide additional comparative references beyond DARCR. Therefore, the MBC-Based and HEED-Based implementations were adapted only to the extent necessary to operate under the same surface-drifting, cluster/backbone reporting scenario considered in the main manuscript.

S2.2. Common Notation Used in the Baseline Implementations

Let $i$denote an ordinary sensor node, c denote a candidate CH, and $u$denote a candidate upstream CH. The Euclidean distance between two nodes $a$and $b$is denoted by $d(a,b)$. The residual energy of node $i$is denoted by E_i, and the initial normalized energy is denoted by E_0. The hop count from a CH u to a base station is denoted by $H(u)$. A smaller $H(u)$ indicates that the upstream candidate is closer to a base station in terms of backbone route depth.

For all normalized quantities used in the MBC-Based baseline, the normalized value is bounded within $[0,1]$. Larger normalized values are preferred unless the term is explicitly used as a penalty. When a score is maximized, the candidate with the largest score is selected. When a communication cost is minimized, the candidate with the smallest cost is selected.

For pairwise mobility estimation, the relative speed between two nodes $a$and $b$is denoted as

$$V_{\mathrm{rel}}(a,b) = \sqrt{(u_a - u_b)^2 + (v_a - v_b)^2}.$$

The estimated connection time between two nodes $a$and $b$under a communication radius R is denoted as $\mathrm{ECT}_R(a,b)$. In this implementation, it is used as a short-term link-persistence indicator under the current drift state. If $d(a,b) > R$, then the link is infeasible and the corresponding candidate is excluded. If $d(a,b) \le R$, a larger estimated connection time indicates a more persistent candidate link. The normalized expected connection time is denoted as

$$\widehat{\mathrm{ECT}}_R(a,b).$$

For member-to-CH association, $R = R_{c1}$. For CH-backbone parent selection, $R = R_{c2}$.

S2.3. MBC-Based Baseline Implementation

S2.3.1. Baseline Rationale

The MBC-Based baseline is used as a mobility-aware clustering comparator. It preserves the core concepts of mobility- and energy-aware CH election, estimated-connection-time-based member association, and mobility-aware backbone parent selection. To make the baseline compatible with the sea-surface reporting scenario considered in this study, the original clustering-oriented design is adapted to the same R_{c1} and R_{c2} based topology-level communication framework used in the main manuscript.

The MBC-Based baseline is divided into three decision stages:

1. CH election;
2. member-to-CH association;
3. CH-backbone parent selection.

S2.3.2. MBC-Based CH Election Score

In the CH-election stage, each alive node is ranked according to normalized residual energy, mobility stability, and BS-oriented reachability. The CH-election score of node $i$is defined as

$$\mathrm{Score}_{\mathrm{CH}}^{MBC-Based}(i) = 0.45\hat{E}_i + 0.20\widehat{M}_i + 0.35\hat{B}_i.$$

Here, $\hat{E}_i$ denotes the normalized residual energy of node i, $\widehat{M}_i$ denotes the normalized mobility-stability score of node i, and $\hat{B}_i$ denotes the normalized BS-oriented reachability score. The residual-energy term gives preference to nodes with higher remaining energy. The mobility-stability term gives preference to nodes with lower drift-induced instability. The BS-oriented reachability term gives preference to nodes that are expected to provide better reachability toward the BS or backbone.

The weights used in the CH-election score are:

Term	weight
Normalized residual energy $\hat{E}_i$	0.45
Normalized mobility stability $\widehat{M}_i$	0.20
Normalized BS-oriented reachability $\hat{B}_i$	0.35

The score is maximized. Therefore, nodes with higher energy, better mobility stability, and better BS-oriented reachability are ranked higher as CH candidates.

S2.3.3. MBC-Based CH-Count Control Rule

The MBC-Based baseline does not impose a fixed final CH count. Instead, the number of CHs is determined by the ranking-based CH-selection process and the uncovered-node promotion rule.

The procedure is as follows:

1. All alive nodes are ranked according to $\mathrm{Score}_{\mathrm{CH}}^{MBC-Based}(i)$.
2. Higher-ranked nodes are considered first as CH candidates.
3. A candidate node is selected as a CH if it improves local coverage feasibility under R_{c1}.
4. After the initial ranking-based selection, if an alive node is not covered by any selected CH within R_{c1}, the highest-ranked feasible uncovered node in that local uncovered region is promoted to CH.
5. The final CH count is therefore produced by the ranking-based selection and uncovered-node promotion process, rather than by enforcing a fixed CH number.

This design avoids imposing an artificial fixed CH count and maintains local coverage feasibility under continuous node drift.

S2.3.4. MBC-Based Member-to-CH Association Score

After CH candidates are selected, each non-CH node chooses a feasible CH within R_{c1}. The member-to-CH association score between ordinary node i and candidate CH c is defined as

$$\text{Score}_{\text{join}}^{MBC-Based}(i,c) = 0.55\widehat{\text{ECT}}_{R_{c1}}(i,c) + 0.25\hat{E}_c - 0.20\frac{d(i,c)}{R_{c1}}, d(i,c) \leq R_{c1}.$$

Here, $\widehat{\text{ECT}}_{R_{c1}}(i,c)$ denotes the normalized expected connection time between node i and candidate CH c, $\hat{E}_c$ denotes the normalized residual energy of candidate CH c, and $d(i,c)/R_{c1}$ denotes the normalized intra-cluster distance penalty.

The weights used in the member-association score are:

Term	weight
Normalized expected connection time $\widehat{\text{ECT}}_{R_{c1}}(i,c)$	0.55
Normalized CH residual energy $\hat{E}_c$	0.25
Normalized distance penalty $d(i,c)/R_{c1}$	−0.20

The score is maximized. Therefore, a member node tends to join a CH that is expected to remain connected for a longer time, has higher residual energy, and is closer in intra-cluster distance.

If no selected CH is reachable within R_{c1}, the node is treated as locally disconnected under the same disconnection-accounting rule used in the comparative evaluation.

S2.3.5. MBC-Based CH-Backbone Parent Selection Score

After member association, CH-backbone routing is constructed under the same R_{c2}-based backbone communication framework used in the main manuscript. A CH may either connect directly to a base station if a base station is reachable within R_{c2}, or select a feasible upstream CH as its backbone parent.

For CH-to-CH backbone construction, an unrouted CH c selects a feasible upstream parent CH u within R_{c2}. The MBC-Based route-selection score is defined as

$$\text{Score}_{\text{route}}^{MBC-Based}(c,u) = 0.55\widehat{\text{ECT}}_{R_{c2}}(c,u) + 0.20\hat{E}_u + 0.25\frac{1}{1+H(u)}, d(c,u) \leq R_{c2}.$$

Here, $\widehat{\text{ECT}}_{R_{c2}}(c,u)$ denotes the normalized expected connection time between CH c and upstream candidate u, $\hat{E}_u$ denotes the normalized residual energy of upstream candidate u, and $1/(1+H(u))$ gives preference to upstream CHs with smaller hop depth.

The weights used in the MBC-Based route-selection score are:

Term	weight
Normalized expected connection time $\widehat{\text{ECT}}_{R_{c2}}(c,u)$	0.55
Normalized upstream residual energy $\hat{E}_u$	0.20
Hop-depth preference $1/(1+H(u))$	0.25

The score is maximized. Thus, the route-selection score balances link persistence, upstream energy reserve, and route-depth control.

A candidate upstream CH is feasible only if:

1. it is alive;
2. it is a CH;
3. it is within R_{c2};
4. it has a finite route to a base station;

5. selecting it does not create an invalid backbone route under the adopted topology-level route-construction rule.

If a CH can directly reach a base station within R_{c2}, the base station is preferentially used as the upstream and the CH hop count is set to 1. Otherwise, the CH selects the feasible upstream CH with the highest $\mathrm{Score}_{\mathrm{route}}^{MBC-Based}(c,u)$.

S2.3.6. MBC-Based Tie-Breaking Rules

All normalized terms in the MBC-Based decision scores are bounded within $[0,1]$, and larger scores are preferred in all three MBC-Based decision stages.

If multiple candidates obtain the same score, ties are resolved using the following order:

1. higher residual energy;
2. longer expected connection time;
3. shorter communication distance;
4. lower hop count, when applicable;
5. smaller node ID.

The lower-hop criterion is applied only in CH-backbone parent selection, where route depth is relevant.

S2.3.7. MBC-Based Control-Plane Accounting

The MBC-Based baseline is evaluated using the same control-plane accounting framework as the other compared methods.

Local clustering announcements, member-association messages, and intra-cluster control transmissions are counted as LowTx. CH-backbone advertisements, CH-to-CH route construction messages, and CH-to-BS / CH-to-CH backbone-control messages are counted as HighTx.

The control-plane cost index is computed consistently with the main manuscript as

$$C_{\mathrm{cp}}(w_H) = \mathrm{LowTx} + w_H \mathrm{HighTx}.$$

When the default reporting-level index is used, $w_H = 256$. Alternative sensitivity weights, including $w_H = 3$ and $w_H = 10$, can be computed from the same raw LowTx and HighTx values.

S2.4. HEED-Based Baseline Implementation

S2.4.1. Baseline Rationale

The adapted HEED-Based baseline is used as an energy- and communication-cost-aware clustering comparator. HEED is a classical distributed clustering protocol in which CH selection is primarily guided by residual energy, while intra-cluster communication cost is used as a secondary clustering criterion.

In this study, the core HEED principle is retained: CH candidacy is primarily determined by residual energy, and member nodes associate with reachable final CHs according to local communication cost. To make the baseline compatible with the sea-surface reporting scenario, CH-to-BS and CH-to-CH backbone construction is added under the same R_{c1} and R_{c2} based topology-level communication framework used in the main evaluation.

S2.4.2. HEED-Based Initial CH Probability

At the beginning of each HEED-Based reclustering process, each alive node first broadcasts a local neighbor-discovery beacon within R_{c1}. This step is used to estimate local communication cost and is counted as a local control transmission, i.e., LowTx.

The initial CH probability of node i is computed as

$$P_{\mathrm{CH}}(i) = \max\left(C_{\mathrm{prob}}\frac{E_i}{E_0}, P_{\min}\right).$$

Here, E_i is the residual energy of node i, E_0 is the initial normalized energy, C_{prob} is the initial CH-probability factor, and $P_{\min}$ is the minimum CH probability.

The parameter values used in the HEED-Based baseline are:

Parameter	Value
C_{prob}	0.01
$P_{\min}$	0.0001
Maximum HEED-Based iterations	6

This probability reflects the residual-energy-first design principle of HEED-Based clustering.

S2.4.3. HEED-Based Communication Cost

The secondary clustering criterion is the intra-cluster communication cost. For a node $i$and a candidate CH c, the communication cost is computed from the normalized intra-cluster distance:

$$C_c(i,c) = \frac{d(i,c)}{R_{c1}}, d(i,c) \le R_{c1}.$$

A smaller $C_c(i,c)$ indicates a lower local communication cost. Candidate CHs outside R_{c1} are infeasible for member association.

S2.4.4. HEED-Based Iteration and CH Finalization Rule

The HEED-Based baseline uses a bounded iterative CH-election process. At each iteration, nodes may announce tentative CH status according to their current $P_{\mathrm{CH}}(i)$. After each iteration, the CH probability is doubled:

$$P_{\mathrm{CH}}(i) \leftarrow \min\left(2P_{\mathrm{CH}}(i),1\right).$$

The process is bounded by six iterations. Tentative CH announcements and final CH announcements are counted as LowTx.

A node becomes a final CH if:

1. it declares itself as a CH during the bounded HEED iteration process; or
2. it remains uncovered by any final CH within R_{c1} at the finalization stage and is promoted to preserve local coverage feasibility.

Therefore, the HEED-Based baseline does not impose a fixed final CH count. The final CH number is determined by the bounded HEED election process and the uncovered-node promotion rule.

S2.4.5. HEED-Based Member Association Rule

After final CHs are determined, each non-CH node joins the reachable final CH with the lowest communication cost:

$$c^* = \arg\min_{c:d(i,c)\le R_{c1}} C_c(i,c).$$

If no final CH is reachable within R_{c1}, the node is treated as locally disconnected under the same disconnection-accounting rule used in the comparative evaluation.

During the HEED finalization stage, if a node is not covered by any final CH within R_{c1}, it is promoted to a final CH to preserve local coverage feasibility. This rule prevents empty local reporting regions during baseline initialization and keeps the HEED-Based baseline comparable to the other cluster-based methods under continuous node drift.

S2.4.6. HEED-Based Backbone Construction

After the final CH set is obtained, CH-to-BS and CH-to-CH backbone construction is performed using the same R_{c2} based topology-level backbone rule used for the other compared methods.

The backbone construction follows these rules:

1. If a final CH can directly reach a base station within R_{c2}, the base station is selected as its upstream and the CH hop count is set to 1.
2. If no base station is directly reachable, the CH searches for a feasible upstream CH within R_{c2}.
3. A candidate upstream CH is feasible only if it is alive, is a final CH, is within R_{c2}, and already has a finite route to a base station.
4. Among feasible upstream CHs, the candidate with the smallest hop count is preferred.
5. If multiple candidates have the same hop count, the candidate with the shorter communication distance is preferred.
6. If a tie still remains, the candidate with higher residual energy is preferred.
7. If a tie still remains, the candidate with the smaller node ID is selected.

Final-CH backbone-status advertisements and backbone route advertisements are counted as HighTx.

S2.4.7. HEED-Based Tie-Breaking Rules

For HEED-Based clustering, ties are resolved in the following order:

1. lower communication cost;
2. shorter communication distance;
3. higher residual energy;
4. smaller node ID.

For HEED-Based backbone construction, ties are resolved in the following order:

1. smaller hop count;
2. shorter CH-to-upstream distance;
3. higher residual energy;
4. smaller node ID.

These tie-breaking rules are applied deterministically to make the baseline reproducible.

S2.4.8. HEED-Based Control-Plane Accounting

The HEED-Based baseline uses the same control-plane accounting framework as the other compared methods.

The following events are counted as LowTx:

1. local neighbor-discovery beacons within R_{c1};
2. tentative CH announcements;
3. final CH announcements;
4. member-to-CH association messages;
5. intra-cluster local control messages.

The following events are counted as HighTx:

1. final-CH backbone-status advertisements;
2. CH-to-CH backbone route advertisements;

3. CH-to-BS backbone-control transmissions;
4. CH-to-CH backbone-control transmissions.

The same control-plane cost index is used:

$$C_{\mathrm{cp}}(w_H) = \mathrm{LowTx} + w_H \mathrm{HighTx}.$$

The default reporting-level index uses $w_H = 256$, while sensitivity values such as $w_H = 3$ and $w_H = 10$ can be computed from the same raw LowTx and HighTx counts.

S2.5. Summary of Baseline Parameter Values

Category	Parameter	Value/Rule
Common communication setting	R_{c1}	5 km
Common communication setting	R_{c2}	20 km
Common energy setting	E_0	5 normalized energy units
MBC-Based CH-election weight	$\hat{E}_i$	0.45
	$\hat{M}_i$	0.20
	$\hat{B}_i$	0.35
MBC-Based association weight	$\widehat{\mathrm{ECT}}_{R_{c1}}(i, c)$	0.55
	$\hat{E}_c$	0.25
	$d(i, c)/R_{c1}$	-0.20
MBC-Based route-selection weight	$\widehat{\mathrm{ECT}}_{R_{c2}}(c, u)$	0.55
	$\hat{E}_u$	0.20
	$1/(1 + H(u))$	0.25
MBC-Based CH count	Final CH number	Determined by ranking-based selection and uncovered-node promotion
HEED-Based CH probability	C_{prob}	0.01
	P_{min}	0.0001
HEED-Based iteration	Maximum iterations	6
HEED-Based CH count	Final CH number	Determined by bounded HEED election and uncovered-node promotion
Control-plane cost	$C_{\mathrm{cp}}(w_H)$	$\mathrm{LowTx} + w_H \mathrm{HighTx}$
Default control-plane index	w_H	256
Sensitivity weights	w_H	3, 10, 256

S2.6. Notes on Fairness of Comparison

The MBC-Based and HEED-Based baselines were not given the proposed event-driven CH-HELP, Node-HELP, route-inheritance repair, or conditional global reclustering mechanisms. This separation is necessary to preserve the methodological distinction between the clustering-oriented baselines and the proposed self-healing topology-maintenance framework.

At the same time, both baselines were adapted to the same surface-drifting reporting scenario by using the same R_{c1} based intra-cluster communication model and the same R_{c2} based CH-backbone construction model. This ensures that the comparison reflects differences in maintenance design rather than differences in deployment area, communication range, base-station placement, environmental forcing, or evaluation metrics.

Therefore, MBC-Based should be interpreted as a mobility-aware clustering comparator, and HEED-Based should be interpreted as an energy- and communication-cost-aware clustering comparator. Their role in the revised manuscript is to supplement the DARCR comparison and to show whether clustering-oriented baseline designs alone can provide the same level of CH-level connectivity preservation as the proposed event-driven maintenance framework under continuous sea-surface drift.
